# Information criterion for approximation of unnormalized densities

**DOI:** 10.1371/journal.pone.0317430

**Published:** 2025-03-17

**Authors:** John Y Choe, Yen-Chi Chen, Nick Terry

**Affiliations:** 1 Department of Industrial and Systems Engineering, University of Washington, Seattle, Washington, United States of America; 2 Department of Statistics, University of Washington, Seattle, Washington, United States of America; Politecnico di Milano, ITALY

## Abstract

This paper considers the problem of approximating an unknown density when it can be evaluated up to a normalizing constant at a finite number of points. This density approximation problem is ubiquitous in statistics, such as approximating a posterior density for Bayesian inference and estimating an optimal density for importance sampling. We consider a parametric approximation approach and cast it as a model selection problem to find the best model in pre-specified distribution families (e.g., select the best number of Gaussian mixture components and their parameters). This problem cannot be addressed with traditional approaches that maximize the (marginal) likelihood of a model, for example, using the Akaike information criterion (AIC) or Bayesian information criterion (BIC). We instead aim to minimize the cross-entropy that gauges the deviation of a parametric model from the target density. We propose a novel information criterion called the cross-entropy information criterion (CIC) and prove that the CIC is an asymptotically unbiased estimator of the cross-entropy (up to a multiplicative constant) under some regularity conditions. We propose an iterative method to approximate the target density by minimizing the CIC. We demonstrate how the proposed method selects a parametric model that well approximates the target density through multiple numerical studies in the Supporting Information.

## 1 Introduction

We consider the problem of finding a parametric approximation of an unknown target density $q^{*}(x),$ from which we cannot sample directly. Instead, we assume the ability to evaluate the unknown non-negative function *r* satisfying $q^{*}(x)=r(x)∕\rho ,$ where *ρ* is an unknown normalizing constant. The function evaluation is expensive enough to worth minimizing it. To refer to this problem in shorthand, we coin a term, the *Boltzmann Approximation problem* (BA problem), owing to its origin in physics. The parametric approximation of $q^{*}$ takes two steps, namely, 1) choosing a parametric family and 2) minimizing the Kullback-Leibler (KL) divergence from a member density in the chosen family to the target density $q^{*}$. The latter is a well-studied optimization problem and the former is an under-explored model selection problem [1]. A criterion for the model selection is the primary contribution of this paper.

The BA problem is ubiquitous in statistics, including three motivating examples:

**Example 1: Simulation-based inference.** Simulation models are widely used to estimate a mean E [v(X)]=ρ. We assume the input **X** follows a *known* nominal density *p* ( *x* ) and run the simulation model to observe a non-negative output, *v* ( *X* ) .

When the simulator is computationally expensive, the importance sampling estimator $\frac{1}{n}\sum_{i=1}^{n}v(X_{i})\frac{p(X_{i})}{q(X_{i})}$ is widely used by sampling ***X**_i_* from *q* instead of *p*. This unbiased estimator has theoretically zero variance if ***X**_i_*, *i* = 1 , … , *n* , follows a density $q(x)=q^{*}(x)=v(x)p(x)∕\rho$ [3]. This optimal density is the approximation target. By defining *r* ( *x* ) = *v* ( *x* ) *p* ( *x* ) , we encounter the BA problem.

**Example 2: Causal inference.** Consider a simple causal inference problem where we define a response *Y *(*A*) under a binary treatment *A* ∈ { 0 , 1 } . Under the potential outcome model [4],

to investigate the causal effect of the treatment on the response, we need to know the densities *p*(*Y*(0)=*y*_0_) and *p*(*Y*(1)=*y*_1_). However, our data only allows us to estimate $p(Y(0)=y_{0}|A=0)$ and $p(Y(1)=y_{1}|A=1)$.

Luckily, Tukey’s factorization allows for approximating the counterfactual density


$$p(Y(0)=y_{0}|A=1)\propto \underset{\lambda (y_{0})}{\underbrace{\frac{P(A=1|Y(0)=y_{0})}{P(A=0|Y(0)=y_{0})}}}p(Y(0)=y_{0}|A=0)$$

and similarly for $p(Y(1)=y_{1}|A=0)$. With a model on *λ*(*y*_0_), we aim to sample from the density implied by the factorization. Defining $q^{*}(\cdot )=p(Y(0)=\cdot |A=1)$ and *r* ( ⋅ ) = *λ* ( ⋅ ) *p* ( *Y* ( 0 ) = ⋅ | *A* = 0 ) yields the BA problem.

**Example 3: Bayesian inference.** The most prominent BA problem is approximating a posterior density for Bayesian inference. Let *r* ( ⋅ ) = *λ* ( ⋅ ) *p* ( *Y* ( 0 ) = ⋅ | *A* = 0 ) be the prior density of the parameter *r* ( ⋅ ) = *λ* ( ⋅ ) *p* ( *Y* ( 0 ) = ⋅ | *A* = 0 ) and $L(\theta |X_{1},\cdots \;,X_{n})$ be the likelihood function. The posterior (density) is $\pi (\theta |X_{1},\cdots \;,X_{n})\propto L(\theta |X_{1},\cdots \;,X_{n})\cdot \pi (\theta )$. In this case, $q^{*}(\cdot )=\pi (\cdot |X_{1},\cdots \;,X_{n})$ is the posterior density and $r(\cdot )=\pi (\cdot )L(\cdot |X_{1},\cdots \;,X_{n})$ is the prior density times the likelihood.

When *r* is computationally light to evaluate, Markov chain Monte Carlo methods have been used extensively, such as the Metropolis-Hastings algorithm that probabilistically accepts or rejects samples. But there is growing interest in the BA problems where evaluating *r* is computationally heavy. In Bayesian inference, *r* may involve computing the likelihood for a large dataset or a complex Bayesian model.

The former case often resorts to the variational inference [5] and the latter case gave rise to the approximate Bayesian computation to bypass evaluating the likelihood function and *r* [6]. For causal inference, if the model involves covariates, we need to sample counterfactual variables several times per each possible covariate value. For simulation-based inference, running a simulator is often computationally expensive (e.g., minutes or hours). In some of these scenarios where we can only afford a relatively small number (e.g., hundreds or thousands) of evaluations of *r* , existing rejection-sampling methods are untenable. This paper assumes we want to use *every* evaluation of *r* to approximate $q^{*}$. As a result, the effort (including computational cost) to better use each evaluation is considered worthwhile and likely negligible compared with the cost of evaluating *r*. Across our simulation studies in the Supporting Information, the average computational time for our algorithm is less than a second per each evaluation of *r*, while evaluating *r* can take minutes or hours in the Examples above.

We are interested in both approximating $q^{*}$ and estimating the normalizing constant *ρ* because *ρ* is, in some applications, even more important to estimate than the density $q^{*}$ itself. *ρ* is the model evidence for Bayesian inference and the estimand for importance sampling. The importance sampling context particularly motivates this study and its emphasis on unbiased estimation of *ρ* (e.g., a rare event probability), which is typically of lesser interest in Bayesian inference.

The existing literature on approximating $q^{*}$ in the BA problem can be generally grouped into parametric and nonparametric approaches [7,8]. This paper considers a class of parametric approaches where we posit a parametric family of densities and find its member closest to $q^{*}$ in terms of the closeness measured by the KL divergence. This parametric framework is very general and includes maximum likelihood estimation. For importance sampling, this framework is used for the so-called cross-entropy method [9].

For this parametric framework, we propose a novel information criterion called the cross-entropy information criterion (CIC). We prove that the criterion is an asymptotically unbiased estimator of the KL divergence (up to a multiplicative constant and an additive constant). We justify that the minimization of the CIC allows for selecting a good family of densities to approximate $q^{*}$ using a limited number of evaluations of *r*.

We also show that the CIC reduces to the Akaike information criterion (AIC)[10] if we have the ability to sample from $q^{*}$ instead of evaluating *r*. Rigorous theoretical analysis of the AIC is a long-standing problem in the literature. Theoretical analyses of information criteria akin to the AIC often impose uniform integrability conditions directly on estimators to express the model complexity penalty in terms of the free parameter dimension *d* . For details, see Conditions A7–A8 in [11], Theorem 1 in [12], a discussion under Eq (7.28) in [13], an approximation from Eqs (2.16) to (2.17) in [14], and more references cited on p. 1157 in[15] and on p. 416 in [17]. For autoregressive models, sufficient conditions for such uniform integrability conditions were established much later than the seminal paper that introduced AIC [10], where the uniform integrability was not established rigorously [17,18]. For general parametric models, establishing general versions of the sufficient conditions remains an open problem. This paper establishes such sufficient conditions for CIC, which is theoretically more general than AIC. Practically, the CIC and AIC are useful for two different classes of model selection problems and hence not interchangeable.

The remainder of this paper is organized as follows. Sect 2 briefly reviews the relevant background. Sect 3 proposes the CIC. Sect 4 explains how the CIC can be used in practice. Proofs, implementation details, and numerical experiments are included in the Supporting Information.

## 2 Background

This section briefly explains the origin of the BA problem and reviews KL divergence, the maximum likelihood estimator (MLE), and the AIC to a) introduce the minimum cross-entropy estimator (MCE), which is a generalized version of MLE, and b) pave the way for generalizing the AIC to the CIC.

The name of the BA problem originates from statistical physics where the nonnegative function *r* ( *x* ) is what we can evaluate and is expressed as $r(x)=e^{-\phi (x)},$ where *ϕ* ( *x* ) is often called the energy of a state *x*. The target density $q^{*}(x)\propto e^{-\phi (x)}$ is called the Boltzmann distribution. Of particular significance in physics is evaluating *ρ* = ∫ *r* ( *x* ) d *x* , called the partition function. The BA problem often has a practically significant consideration: evaluating *r* is (computationally) expensive, so we want to make use of all evaluations.

KL divergence is commonly used to gauge a difference between two distributions in statistical inference. Consider two probability measures, *Q*^∗^ and *Q*, on a common measurable space such that *Q*^∗^ is absolutely continuous with respect toQ( written *Q*^∗^≪*Q*).

The MLE is a prominent example of using the KL divergence. When the data ***X***_1,…,_***X****_n_* are drawn from an unknown *Q*^∗^ (note that such direct sampling is not possible in the BA problem), we can approximate *Q*^∗^ by a member in a parametric family$\left\{Q_{\theta }:\theta \in \Theta_{d}\subset {\mathbb{R}}^{d}\right\}$ by minimizing the KL divergence from *Q****_θ_*** to *Q*^∗^ over $\theta \in \Theta_{d}$. Suppose $Q^{*}\ll Q_{\theta }$ for all $\theta \in \Theta_{d}$ so that the KL divergence is well defined over $\Theta_{d}$. Also, for a dominating measure *μ* (e.g., counting or Lebesgue measure), suppose $Q_{\theta }\ll \mu$ for all $\theta \in \Theta_{d}$ and $Q^{*}\ll \mu$ so that densities $q_{\theta }=dQ_{\theta }∕d\mu$ and $q^{*}=dQ^{*}∕d\mu$ exist. Then, the KL divergence is


D (Q∗||Qθ)= ∫ q∗log ⁡  (q∗qθ)dμ= ∫ q∗log ⁡ q∗dμ−∫ q∗log ⁡ qθdμ. (1)

Note that only the second term in (1), called cross-entropy, depends on *μ*. Therefore, minimizing the KL divergence over $\theta \in \Theta_{d}$ is equivalent to minimizing the cross– entropy over $\theta \in \Theta_{d}$. Because $q^{*}$ is unknown, the cross-entropy should be estimated based on $X_{1},\ldots ,X_{n}∼Q^{*}$. An unbiased, consistent estimator of the cross-entropy is


−1n∑i=1n log ⁡ qθ (Xi). (2)

The MLE of *μ*, denoted by ${\hat{\theta }}_{n}$, is the minimizer of the cross-entropy estimator in (2).

Another example of using the KL divergence or cross-entropy is the Akaike information criterion (AIC) [10]. (We continue to use the same notations as above.) As the dimension *d* of the free parameter space $\Theta_{d}$ (or equivalently, the model degrees of freedom) increases, the bias of $Q_{{\hat{\theta }}_{n}}$ approximating *Q*^∗^ will reduce. To compare the different approximating distributions (or models), we could use a plug-in estimator


−1n∑i=1n log ⁡ qθ^n (Xi) (3)

of the cross-entropy. But, this causes an overfitting problem because of the downward bias created from using the data twice (once for ${\hat{\theta }}_{n}$ and another for estimating the cross-entropy).

The AIC remedies this issue by correcting the asymptotic bias of the estimator in (3). The AIC is defined (up to a multiplicative constant) as


−1n∑i=1n log ⁡ qθ^n (Xi)+dn, (4)

where the bias correction term *d* ∕ *n* penalizes the model complexity, balancing it with the goodness-of-fit represented by the first term. Minimizing the AIC is minimizing an asymptotically unbiased estimator of the cross-entropy. Therefore, both the MLE and AIC aim at minimizing the cross-entropy from an approximate distribution *Q****_θ_*** to *Q*^∗^.

Hereafter, we continue to use the notations *Q****_θ_*** and *Q*^∗^ for the approximate and target distribution, respectively. But, note that in the BA problem we cannot generate/sample data directly from *Q*^∗^. Instead, we can evaluate *r*, which is equal to $q^{*}$ up to an unknown normalizing constant *ρ*, at a limited number of points.

## 3 Cross-entropy information criterion for the BA problem

We first introduce an iterative approximation of the cross-entropy in the BA problem, which is suitable for parameter estimation for a fixed-dimension parametric family, in Sects 3.1 and 3.2. In Sect 3.3, we then present the CIC, which can be used for model selection between families with different parameter dimensions. These concepts are combined to yield a sequential algorithm for parameter estimation and model selection. As part of the development, we first define the algorithm in a form which estimates parameters using data from only one stage at a time. In Sect 3.4, we then provide a practical version of the algorithm which uses the cumulative data from all stages.

### 3.1 Approximate Cross-Entropy (ACE) for the BA problem

Analogous to the MLE and AIC, our approximation task in this paper considers minimizing the KL divergence (or cross-entropy) from a parametric distribution *Q****_θ_*** to the target distribution *Q*^∗^ over $\theta \in \Theta_{d}$ (to well-define the KL divergence, hereafter assume $Q^{*}\ll Q_{\theta }$ for all $\theta \in \Theta_{d}$) when the target density $q^{*}$ is proportional to a nonnegative function *r*, i.e., $q^{*}=r∕\rho$ for a positive unknown constant *ρ* = ∫ *r* d *μ*. Recall that in the BA problem, we cannot directly sample from *Q*^∗^ but can evaluate *r* at a limited number of points. Minimizing the KL divergence in (1) over *r* is equivalent to minimizing the cross– entropy - ∫q∗log ⁡ qθdμ=−1ρ ∫  ⁡ rlog ⁡ qθdμ and equivalent to minimizing


C(θ):=−∫ rlog ⁡ qθdμ, (5)

which is unknown in practice because *r* can be evaluated only at observed data points. Using importance sampling, we approximate *r* in (5) by an unbiased and consistent estimator


C¯η(θ):=−1n∑i=1nr(Xi)qη (Xi) log ⁡ qθ (Xi), (6)

where $X_{1},\ldots ,X_{n}∼Q_{\eta }$ with any parameter $\eta \in \Theta_{d}$ as long as the support of *q****_η_*** covers the support of $q^{*}$ regardless of any model misspecification (i.e., $Q^{*}\notin \{Q_{\eta }|\eta \in \Theta_{d}\}$) in practice. Because the estimator in (6) is to approximate the cross-entropy in the BA problem, we call it the *approximate cross-entropy* (ACE).

Therefore, by minimizing ${\bar{C}}_{\eta }(\theta )$ in (6) over $\theta \in \Theta_{d}$, we can approximately minimize the KL divergence (or cross-entropy) from *Q****_θ_*** to *Q*^∗^. Thus, we call


θ^n:=arg ⁡ min ⁡ θ∈ΘdC¯η(θ) (7)

the *minimum cross-entropy estimator* (MCE) because it minimizes the ACE. Note that if the random sample is directly drawn from the target distribution (i.e., $X_{1},\ldots ,X_{n}∼Q_{\eta }=Q^{*}$), then the MCE reduces to the MLE because minimizing (6) is equivalent to minimizing (2) due to $r∕q^{*}=\rho$. More properties of the MCE (e.g., consistency, asymptotic normality, limiting behavior) are characterized in Appendix A.

In the importance sampling literature, the density *q****_η_*** in (6) is called the importance sampling (or proposal) density and $q^{*}$ the optimal importance sampling density. The density $q^{*}$ is optimal because the variance of the following importance sampling estimator of *ρ* is reduced to zero if $X_{1},\ldots ,X_{n}$ are drawn from $q_{\eta }=q^{*}$:


$$\begin{array}{ll} {\hat{\rho }}_{\text{IS}} & =\frac{1}{n}\overset{n}{\underset{i=1}{\sum }}\frac{r\;\left(X_{i}\right)}{q_{\eta }\;\left(X_{i}\right)}.\; \end{array}$$ (8)

In practice, $q^{*}$ is unknown and thus approximated by *q****_η_***. Therefore, finding *q****_η_*** closest to $q^{*}$ is of primary interest.

### 3.2 Iterative procedure for minimizing the ACE

A bad choice of sampling density *q****_η_*** may lead to a large variance of the ACE ${\bar{C}}_{\eta }(\theta )$ in (6). Inspired by the cross-entropy method [9], we propose to update *η* iteratively by the closest estimate of ***θ***^∗^ as described in Box 1. We use the same sample size *n* for each iteration for notational simplicity without loss of generality. Later, Sect 3.4 will discuss a more efficient algorithm that uses data in a cumulative fashion.

**Box 1: Iterative procedure for approximating *Q* ^∗^ by minimizing the estimator of cross– entropy from a parametric distribution *Q_θ_* to *Q* ^∗^.**

Iterative procedure for approximating*Q*^∗^

Inputs: iteration counter *t* = 1, the number of iterations *τ*, the sample size *n*, and the initial parameter ${\hat{\theta }}_{n}^{\left(0\right)}=\eta \in \Theta_{d}$.

1. Sample $X_{1}^{\left(t-1\right)},\ldots ,X_{n}^{\left(t-1\right)}$ from $Q_{{\hat{\theta }}_{n}^{\left(t-1\right)}}$.
2. Find the MCE θ^n(t):=arg ⁡ min ⁡ θ∈ΘdC¯θ^n(t−1)(θ), where
  C¯θ^n(t−1)(θ):=−1n∑i=1nr (Xi(t−1))qθ^n(t−1) (Xi(t−1)) log ⁡ qθ (Xi(t−1)). (9)
3. If *t* = *τ*, output the approximate distribution $Q_{{\hat{\theta }}_{n}^{\left(\tau \right)}}$. Otherwise, increment *t* by 1 and go to Step 1.

An important property of the procedure in Box 1 is that the iterative update of *η* by the best estimate of ***θ***^∗^ leads to an information criterion that mimics the AIC, as detailed in the next section. If *η* is fixed, we will not obtain such a nice property.

The iterative approach can be used to estimate *ρ*, the normalizing constant, by modifying the estimator in (8) as follows:


$$\begin{array}{ll} {\hat{\rho }}^{\left(t-1\right)} & =\frac{1}{n}\overset{n}{\underset{i=1}{\sum }}\frac{r\;\left(X_{i}^{\left(t-1\right)}\right)}{q_{{\hat{\theta }}_{n}^{\left(t-1\right)}}\;\left(X_{i}^{\left(t-1\right)}\right)}.\; \end{array}$$ (10)

Furthermore, if $q_{{\hat{\theta }}_{n}^{\left(t-1\right)}}=q^{*}$, the estimator in (10) is the optimal importance sampling estimator having zero variance. Because the iterative procedure refines $q_{{\hat{\theta }}_{n}^{\left(t-1\right)}}$ to be closer to $q^{*}$, ${\hat{\rho }}^{\left(t-1\right)}$ will generally have a smaller variance as *t* gets larger.

Suppose the time complexity for sampling a single observation *r* ( *X* ) based on **X** $∼Q_{\theta }$ is *O* ( *a* ) and the time complexity of optimizing the MCE is *O* ( *b* ) . Then the total time complexity for running the algorithm in Box 1 for *τ* iterations is *O* ( *τ* ( *na* + *b* ) ) . So when the sample size *n* is large and/or evaluating *r* ( ⋅ ) is expensive, most of the cost is from sampling rather than the optimization. So we are allowed to use a more time-consuming optimization program to find the MCE without increasing too much of the total computational cost. In particular, when we use the EM algorithm to find the MCE (see Appendix B for the algorithm details), the time-complexity *O* ( *b* ) = *O* ( *K* ) , where *K* is the number of iterations in the EM algorithm. In this case, setting *K* = *O* ( *n* ) will maintain the total time complexity to be *O* ( *τn* ) .

As for the space complexity, Step 1 of the algorithm in Box 1 requires *O* ( *n* ) to store sampled values. Step 2’s space complexity depends on the optimization algorithm choice. When we use the EM algorithm, its expectation and maximization steps only need to store function values evaluated for *n* observations at the last EM iteration’s parameters, not the entire EM iteration history. As a result, the required space for the *t^th^* iteration isO(dn), whered is the dimension of *n*. Over *τ* iterations in Box 1, only the previous iteration’s information needs to be kept, resulting in the total space complexity for Step 2 to remain at *O* ( *dn* ) . In practice, because *r* ( ⋅ ) is expensive to evaluate, storing all its evaluations across *τ* iterations is sensible, although not required. Considering this addition of *O* ( *τn* ) , the total space complexity is *O* ( *dn* + *τn* ) .

### 3.3 Cross-entropy information criterion (CIC)

To simplify the model complexity penalty term in the AIC, Akaike [10] assumes that the true data-generating distribution belongs to the parametric distribution family being considered. We make a similar assumption $Q^{*}=Q_{\theta^{*}}$ (i.e., assumption (A1) in Appendix A) to simplify the asymptotic bias of ${\bar{C}}_{{\hat{\theta }}_{n}^{\left(t-1\right)}}\;\left({\hat{\theta }}_{n}^{\left(t\right)}\right)$ in estimating $C\left({\hat{\theta }}_{n}^{\left(t\right)}\right)$.

In what follows we describe our main result as Theorem 1 that quantifies the asymptotic bias. The assumptions and proof are deferred to Appendix A.

**Theorem 1** (Asymptotic bias of ${\bar{C}}_{{\hat{\theta }}_{n}^{\left(t-1\right)}}\left({\hat{\theta }}_{n}^{\left(t\right)}\right)$ in estimating $C\left({\hat{\theta }}_{n}^{\left(t\right)}\right)$). *Suppose that assumptions (A1-6) and (B1-5) hold. Then*


$$\begin{array}{llll} E\;\left[{\bar{C}}_{{\hat{\theta }}_{n}^{\left(t-1\right)}}\;\left({\hat{\theta }}_{n}^{\left(t\right)}\right)-C\;\left({\hat{\theta }}_{n}^{\left(t\right)}\right)\right] & =-\rho \frac{d}{n}+o\;\left(\frac{1}{n}\right)\; & & \; \end{array}$$

*for each* *t* = 2 , … , *τ*.

In the above theorem, there are two sources of randomness in the expectation. The first one is ${\bar{C}}_{{\hat{\theta }}_{n}^{\left(t-1\right)}}\;\left(\cdot \right)$, and the second is ${\hat{\theta }}_{n}^{\left(t\right)}$. The bias occurs because these two are dependent, and this is the key insight leading to the derivation of an information criterion akin to AIC.

The asymptotic bias, − *ρd* ∕ *n*, is proportional to the free parameter dimension *d* of the parameter space $\Theta_{d}$, similar to the penalty term of the AIC in (4). In practice, $\rho =E_{\mu }\;\left[r\right]$ is unknown, but we can use a consistent estimator of *ρ* to estimate the asymptotic bias, such as the estimator in (10).

As a bias-corrected estimator of the cross-entropy $C\;\left({\hat{\theta }}_{n}^{\left(t\right)}\right)$ (up to a multiplicative constant), we define the *cross-entropy information criterion* (CIC) as follows:

**Definition 1 (Cross-entropy information criterion (CIC)).**

$$\begin{array}{ll} {CIC}^{\left(t\right)}(d)={\bar{C}}_{{\hat{\theta }}_{n}^{\left(t-1\right)}}\;\left({\hat{\theta }}_{n}^{\left(t\right)}\right)+\hat{\rho }\frac{d}{n} & \; \end{array}$$ (11)

for *t* = 1 , … , *τ* , where θ^n(t):=arg ⁡ min ⁡ θ∈ΘdC¯θ^n(t−1)(θ) with ${\bar{C}}_{{\hat{\theta }}_{n}^{\left(t-1\right)}}(\cdot )$ in (9). $\hat{\rho }$ is a consistent estimator of *ρ*, such as the estimator in (10).

We note that the CIC reduces to the AIC up to an additive $o_{p}(1∕n)$ if the samples are all drawn from the target distribution, that is, $X_{1}^{\left(t-1\right)},\ldots ,X_{n}^{\left(t-1\right)}∼Q_{{\hat{\theta }}_{n}^{\left(t-1\right)}}=Q^{*}$ for *t* = 1 , … , *τ* in Box 1. If so, the first term of the CIC in (11) becomes


C¯θ^n(t−1) (θ^n(t)):=−1n∑i=1nr (Xi(t−1))qθ^n(t−1) (Xi(t−1)) log ⁡ qθ^n(t) (Xi(t−1)) (12)

=−1n∑i=1nr (Xi(t−1))q∗ (Xi(t−1)) log ⁡ qθ^n(t) (Xi(t−1)) (13)

=−ρn∑i=1n log ⁡ qθ^n(t) (Xi(t−1)), (14)

because $q_{{\hat{\theta }}_{n}^{\left(t-1\right)}}=q^{*}$ in (12) and $r∕q^{*}=\rho$ in (13). Plugging the expression in (14) into the CIC in (11) shows that the CIC is equal to *ρ* times the AIC in (4) up to an additive $o_{p}(1∕n)$. Note that unless the exact sampling from the target distribution is possible, the CIC remains different from the AIC. Thus, it is generally indefensible to use the AIC in lieu of the CIC for the model selection under consideration in this paper.

The asymptotic bias expression in Theorem 1 holds only for *t* ≥ 2, because when *t* = 1, the initial sample is drawn from $Q_{{\hat{\theta }}_{n}^{\left(0\right)}}=Q_{\eta }$, which is not a distribution converging to *Q****_θ_***^∗^. Regardless, in practice, one may still use the CIC to select a reasonable parameter dimension *d* at the first iteration (*t* = 1).

### 3.4 The CIC based on cumulative data

If we use the equal sample size *n* for each iteration, the model dimension *d* for later iterations may vary only a little from the earlier iterations. Alternatively, we can aggregate the samples gathered through iterations to obtain a cumulative version of the CIC as discussed in this subsection.

In the *t^th^* iteration, the cumulative version uses all the observed data up to the current iteration to estimate *d*, instead of using only the current iteration’s data $X_{1}^{\left(t-1\right)},\ldots ,X_{n}^{\left(t-1\right)}∼Q_{{\hat{\theta }}_{n}^{\left(t-1\right)}}$ (recall Box 1). The benefit of the cumulative version is the tendency of the aggregated estimator of *d* to have a smaller variance than the non– aggregated estimator ${\bar{C}}_{{\hat{\theta }}_{n}^{\left(t-1\right)}}(\theta )$ in (9). This approach, in turn, can reduce the variance of the MCE as well, which minimizes the aggregated estimator of *d*.

For more flexibility, we can allocate a different sample size for each iteration, that is, *n_t_* for the *t^th^* iteration, *t* = 0 , 1 , … , *τ* (for example, a large *n*_0_ for the initial sample to broadly cover the support of *Q****_η_*** and equal sample sizes $n_{1}=\ldots =n_{\tau }$ for the later iterations). Then, we can find the MCE


θ^(t):=arg ⁡ min ⁡ θ∈ΘdC¯(t−1)(θ), (15)

where the aggregated estimator of *t* = 0 , 1 , … , *τ* is denoted as


C¯(t−1)(θ):=1∑s=0t−1ns ∑s=0t−1nsC¯θ^(s)(θ)=−1∑s=0t−1ns ∑s=0t−1 ∑i=1nsr (Xi(s))qθ^(s) (Xi(s)) log ⁡ qθ (Xi(s)) (16)

for *t* = 1 , … , *τ* with ${\hat{\theta }}^{\left(0\right)}:=\eta$. Note that ${\bar{C}}^{\left(t-1\right)}(\theta )$ in (16) is an unbiased estimator of *t* = 1 , … , *τ*.

By using all data gathered up to the *t*^th^ iteration, we can determine the model parameter dimension *d* at the *t*^th^ iteration with the following CIC:

**Definition 2 (CIC: Cumulative version).**

$$\begin{array}{ll} {\bar{CIC}}^{\left(t\right)}(d)={\bar{C}}^{\left(t-1\right)}\;\left({\hat{\theta }}^{\left(t\right)}\right)+\hat{\rho }\frac{d}{\overset{t-1}{\underset{s=0}{\sum }}n_{s}} & \; \end{array}$$ (17)

for *t* = 1 , … , *τ* , where θ^(t):=arg ⁡ min ⁡ θ∈ΘdC¯(t−1)(θ) in (15). $\hat{\rho }$ is a consistent estimator of *ρ*, such as the estimators in (18) and (19).

As *t* increases, the accumulated sample size $\sum_{s=0}^{t-1}n_{s}$ increases so that the free parameter dimension *d* can increase. Thus, the cumulative version of the CIC allows the use of a highly complex model if it can better approximate *Q*^∗^.

Note that the cumulative version of the CIC in (17) is motivated by the non-cumulative version in (11), but it remains unclear if a result similar to Theorem 1 will appear in this case. The major challenge is that the latest iteration depends on information from all previous iterations, so the analysis on the bias becomes more complicated.

As a consistent and unbiased estimator of *ρ* (akin to the estimators in (6) and (8)), we can use


$$\begin{array}{ll} {\hat{\rho }}^{\left(0\right)} & =\frac{1}{n_{0}}\overset{n_{0}}{\underset{i=1}{\sum }}\frac{r\;\left(X_{i}^{\left(0\right)}\right)}{q_{{\hat{\theta }}^{\left(0\right)}}\;\left(X_{i}^{\left(0\right)}\right)}\; \end{array}$$ (18)

at the 1*^st^* iteration. At the *t^th^* iteration for *t* = 2 , … , *τ*, we can use


$$\begin{array}{ll} {\hat{\rho }}^{\left(t-1\right)} & =\frac{1}{\overset{t-1}{\underset{s=1}{\sum }}n_{s}}\overset{t-1}{\underset{s=1}{\sum }}\overset{n_{s}}{\underset{i=1}{\sum }}\frac{r\;\left(X_{i}^{\left(s\right)}\right)}{q_{{\hat{\theta }}^{\left(s\right)}}\;\left(X_{i}^{\left(s\right)}\right)},\; \end{array}$$ (19)

where we do not use the data $X_{1}^{\left(0\right)},\ldots ,X_{n_{0}}^{\left(0\right)}$ from the initial distribution $Q_{{\hat{\theta }}^{\left(0\right)}}=Q_{\eta }$ because they could potentially increase the variance of the resulting estimator if *Q****_η_*** is too different from *Q*^∗^. The estimator in (19) is an importance sampling estimator of $\rho =E_{\mu }\;\left[r\right]$. The potential for the increased variance has been well studied in the importance sampling literature, including defensive techniques [19,20]. Owen and Zhou’s method (SEIS) is implemented and tested in S2 Additional experiments (Interested readers can find cemSEIS.py in our Python package linked in S3 Code). Note also that one can choose the initial distribution (denoted by $Q_{{\hat{\theta }}^{\left(0\right)}}=Q_{\eta }$ thus far) to be any distribution whose support covers the support of *Q*^∗^ to keep the estimator in (18) unbiased, although a judicious choice can reduce the estimator’s variance. Future research may investigate how to assign greater weights on newer observations to improve the estimation depending on the asymptotic behavior of $q_{{\hat{\theta }}^{\left(s\right)}}$ approaching $q^{*}$.

## 4 Application of the cross-entropy information criterion

This section details how the cumulative version of CIC can be useful in practice. Henceforth, CIC refers to the cumulative version (Definition 2), not the non-cumulative version (Definition 1), unless specified otherwise. We first present how the CIC can help choose the number of components, *k*, for a mixture model in conjunction with an expectation-maximization (EM) algorithm that finds the MCE for a given *k*. Then, we present the summary of how to use the CIC to iteratively approximate a target distribution. In Appendix C, we provide numerical examples to illustrate the use of the CIC for approximating the optimal importance sampling density and the posterior density in Bayesian inference. Additional numerical experiments are included in S2 Additional experiments, which investigate when the CIC-based importance sampling using a Gaussian mixture model works well or not. Interested readers are also referred to the work of [21], which applies the CIC-based importance sampling to stochastic simulation models, where the evaluation of *r* is stochastic (i.e., *r* ( *x* ) is not a function, but it follows a distribution that depends on *x*).

### 4.1 Simulation: Mixture model and an EM algorithm

To approximate a target distribution, we consider a parametric mixture model with a parameter dimension *d*. Parametric mixture models are often used to approximate a posterior density for Bayesian inference [5] and an optimal importance sampling density [3,22,23]. The density approximation quality hinges on the number of mixture components, *k* (or equivalently, the parameter dimension *d*). Prior studies either assume that *k* is given [3,22] or use a rule of thumb to choose *k* based on “some understanding of the structure of the problem at hand” [23].

We can use the CIC to select *k* for any parametric mixture model, considering various parametric component families. For example, exponential families are especially convenient because the MCE can be found by using an expectation-maximization (EM) algorithm. In this paper, we use the Gaussian mixture model (GMM) for illustration. Appendix B details our version of the EM algorithm to find the MCE. Future research may investigate computationally more efficient methods based on the recent advancement of distributed computing and gradient-based methods [2].

**Fig 1:**
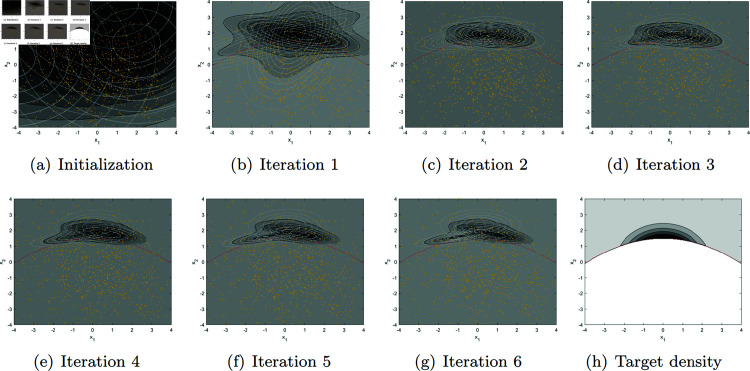
Illustration of our EM algorithm (for *k* = 3 at *t* = 1) that updates a randomly initialized density in (a) through Iterations 1–6 in (b)–(g) to approximate the unknown target density in (h). *Yellow dots* in (a)–(g) are the 2-dimensional pilot data $X_{i}^{\left(0\right)},i=1,\ldots$, 1000 sampled from the initial distribution $Q_{{\hat{\theta }}^{\left(0\right)}}=Q_{\eta }$. Gray-scale filled contour plots represent the Gaussian mixture density with *k* = 3 components in (a)–(g) and the target density in (h). *White contour line plots* in (a)–(g) represent the three component densities of the Gaussian mixture density. *Red dashed line* is the reference line that marks the shape of the target density in (h). The target density is the optimal importance sampling density in the structural safety example (with *b* = 1 . 5) in Appendix C.

Fig 1 illustrates our EM algorithm in action for the first outer iteration (*t* = 1), where a GMM (gray-scale filled countour plot) with three component densities (white countour lines) is updated over EM iterations to approximate an unknown target density. We can see that in contrast to the conventional EM algorithm that maximizes the likelihood of a model (i.e., goodness-of-fit) to approximate the distribution of observed data, our algorithm uses the data (yellow dots), $X_{1}^{\left(0\right)},\ldots ,X_{1000}^{\left(0\right)}∼Q_{\eta }$, to estimate and minimize the cross-entropy from the approximate density to the target density. Out of the 1000 observations (yellow dots) in Fig 1(a) (note that the same data are plotted in (b)–(g) as well), only the small portion of them that fall *above* the red dashed line contribute to the cross-entropy estimate (in (16) because *r* ( *x* ) is zero *below* the red dashed line for the structural safety example in Appendix C). See Appendix D for more details of the EM algorithm (including its parameter initialization strategy based on [16]) implemented for the structural safety example in Appendix C.

### 4.2 Summary of the CIC-based distribution approximation procedure

This subsection summarizes how we can use the CIC to approximate a target distribution in practice. Using the EM algorithm in Sect 4.1 for different *k*’s (or *d*’s) in the *t^th^* iteration for *t* = 1 , … , *τ*, we can find the MCE in (15) and calculate the CIC in (17). At the minimum of the CIC, we can then find the best number of components, $k^{*(t)}$ ( or the best model dimension $d^{*(t)})$ to use in the *t^th^* iteration.

**Fig 2:**
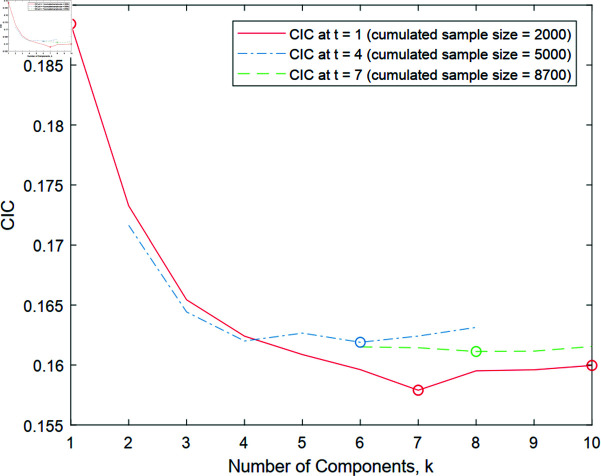
Plot of the CIC (cumulative version), ${\bar{\text{CIC}}}^{\left(t\right)}(d)$, in (17) versus the number of components *k*, which determines the model dimension *d*, of the Gaussian mixture model. As the iteration counter *t* increases from 1 (red solid line) to 4 (blue dash-dot line) to 7 (green dashed line), the CIC is calculated using a larger sample. The CIC is minimized at *k* = 7 for *t* = 1, *k* = 6 for *t* = 4, and *k* = 8 for *t* = 7 in this example. The circles in the plot correspond to the approximate densities shown in Fig 3(a)–3(e).

The CIC tends to decrease and then slowly increase as *d* increases, subject to the randomness of the data. Fig 2 shows such a pattern, where *k* is the number of mixture components in the GMM with unconstrained means and covariances. Note that *k* is proportional to the free parameter dimension *d* = ( *k* − 1 ) + *k* ( *p* + *p* ( *p* + 1 ) ∕ 2 ) , with *p* denoting the dimension of the GMM density support.

Within the *t^th^* iteration, we calculate the CIC over *k* as shown in Fig 2 for *t* = 1, 4, 7 for the structural safety example in Appendix C. As the iteration counter *t* increases, ${\bar{\text{CIC}}}^{\left(t\right)}(d)$ in (17) uses a larger sample that accumulated data over iterations to more accurately estimate the cross-entropy.

**Fig 3:**
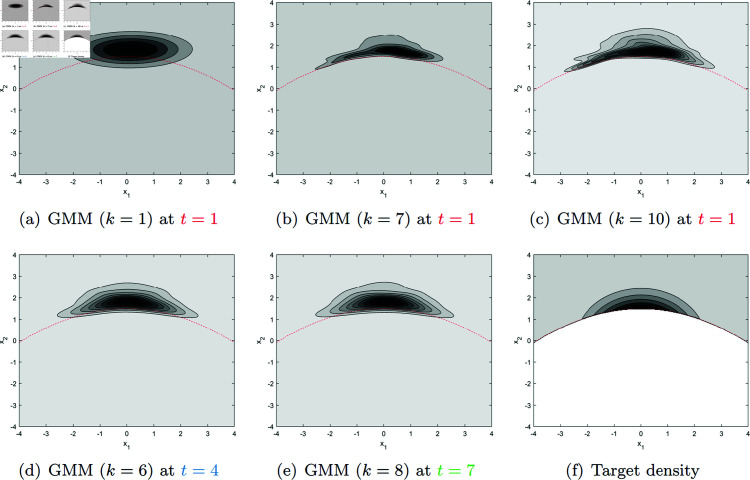
Gaussian mixture models (GMMs) with *k* components in (a)–(e) correspond to the circles in Fig 2 and are compared with the target density in (f), which is the optimal importance sampling density in the structural safety example (with *b* = 1 . 5) in Appendix C.

At *t* = 1, the GMM with *k* = 7 in Fig 3(b) achieves the minimum CIC (as shown in Fig 2), while *k* = 1 and *k* = 10 result in seemingly over-simplified and over-complicated densities in Figs 3(a) and 3(c), respectively, for the given sample size, 1000 (note that the effective sample size is much smaller because only a small portion of the data fall above the red dashed line as explained earlier with Fig 1). A good choice of *k* (neither too small nor too large) for the given sample size at the current iteration helps subsequent iterations by preventing sampling from an overly simplified/complicated distribution that could misguide the later iterations. Thus, it is beneficial to refine the approximate distribution *proportionally* (neither too much nor too little) for the given data size as guided by the CIC. Over iterations, sampled data (yellow dots in Fig 1) should increasingly cover the entire support of the target density. The CIC-minimizing densities at *t* = 4 in Fig 3(d) and *t* = 7 in Fig 3(e) capture the overall shape of the target density in Fig 3(f).

Box 2 summarizes the CIC-based distribution approximation procedure. Note that in addition to approximating the target distribution *Q*^∗^, if we want to estimate a quantity of interest such as *ρ*, we can sample $X_{1}^{\left(\tau \right)},\ldots ,X_{n_{\tau }}^{\left(\tau \right)}∼Q_{{\hat{\theta }}^{\left(\tau \right)}}$ and use the estimator such as ${\hat{\rho }}^{\left(\tau \right)}$ in (19). The numerical examples in the Supporting Information use this additional step.

**Box 2: CIC-based approximation of the target distribution*Q* ^∗^.**

CIC-Based Approximation of the Target Distribution *Q*^∗^

Inputs: iteration counter *t* = 1, the number of iterations *τ*, the sample size per iteration $n_{t},t=1,\ldots ,\tau$, the initial parameter dimension *d*^(0)^, and the initial parameter ${\hat{\theta }}^{\left(0\right)}=\eta \in \Theta_{d^{\left(0\right)}}$.

1. Sample $X_{1}^{\left(t-1\right)},\ldots ,X_{n_{t-1}}^{\left(t-1\right)}∼Q_{{\hat{\theta }}^{\left(t-1\right)}}$.
2. Find the best model dimension d∗(t):=arg ⁡ min ⁡ d≥1CIC¯(t)(d) to use, where ${\bar{\text{CIC}}}^{\left(t\right)}(d):={\bar{C}}^{\left(t-1\right)}\;\left({\hat{\theta }}^{\left(t\right)}\right)+\hat{\rho }\frac{d}{\sum_{s=0}^{t-1}n_{s}}$ is in (17) with the MCE θ^(t):=arg ⁡ min ⁡ θ∈ΘdC¯(t−1)(θ) in (15). $\hat{\rho }$ is a consistent estimator of *ρ*, such as the estimators in (18) and (19).
3. If *t* = *τ*, output the approximate distribution $Q_{{\hat{\theta }}^{\left(\tau \right)}}$. Otherwise, increment *t* by 1 and go to Step 1.

## 5 Conclusion

This paper proposed the cross-entropy information criterion (CIC) to find a parametric density that has the asymptotically minimum cross-entropy to a target density to approximate. The CIC is the sum of two terms: an estimator of the cross-entropy (up to a multiplicative constant) from the parametric density to the target density, and a model complexity penalty term. Under certain regularity conditions, we proved that the penalty term corrects the asymptotic bias of the first term in estimating the true cross-entropy. Empirically, we demonstrated that minimizing the CIC leads to a density that well approximates a target density.

The CIC allowed us to develop a principled algorithm to near-automatically approximate an unknown density that can be evaluated up to a normalizing constant at a limited number of points. The necessary manual “tuning” in practice is minimal as it pertains primarily to the selection of the initial sampling distribution, $Q_{{\hat{\theta }}^{\left(0\right)}}=Q_{\eta }$. It can be chosen judiciously based on a priori knowledge about where *r* is expected to be large (or at least, non-zero) once one determines the mixture component distribution (e.g., Gaussian vs. something else) and the algorithm for minimizing the CIC (e.g., EM algorithm). This paper’s CIC-based algorithm is made publicly available as the first off-the-shelf software package for solving the BA problem.

## Supporting information

S1 Appendices It includes Appendix A (Assumptions and Proofs), Appendix B (EM Algorithm for Minimizing the Cross-Entropy), Appendix C (Numerical Experiments: Importance Sampling and Bayesian Inference), and Appendix D (Implementation Details of Numerical Experiments).
(PDF)

S2 Additional experiments It includes additional numerical experiments that investigate the empirical performance of the CIC-based importance sampling through two numerical examples and one case study
(PDF)
**S3 Code.** The code for reproducing all experimental results in the paper is publicly available as a Python package on https://pypi.org/project/cicriterion/. Its archived version’s DOI is doi: 10.5281/zenodo.13901261

## References

[1] Chan JCC, Kroese DP. Improved cross-entropy method for estimation. Stat Comput. 2011;22(5):1031–40. doi: 10.1007/s11222-011-9275-7

[2] Chen Y-C. Statistical inference with local optima. J Am Statist Assoc. 2022;118(543):1940–52. doi: 10.1080/01621459.2021.2023550

[3] Botev ZI, Kroese DP, Rubinstein RY, L’Ecuyer P. The cross-entropy method for optimization. In: Govindaraju V, Rao C R, editors. Machine learning: theory and applications, vol. 31, Chennai: Elsevier. 2013. p. 35–59.

[4] Rubin DB. Causal inference using potential outcomes. J Am Statist Assoc. 2005;100(469):322–31. doi: 10.1198/016214504000001880

[5] Blei DM, Kucukelbir A, McAuliffe JD. Variational inference: a review for statisticians. J Am Statist Assoc. 2017;112(518):859–77. doi: 10.1080/01621459.2017.1285773

[6] Cranmer K, Brehmer J, Louppe G. The frontier of simulation-based inference. Proc Natl Acad Sci U S A. 2020;117(48):30055–62. doi: 10.1073/pnas.1912789117 32471948 PMC7720103

[7] Choe Y, Byon E, Chen N. Importance sampling for reliability evaluation with stochastic simulation models. Technometrics. 2015;57(3):351–61. doi: 10.1080/00401706.2014.1001523

[8] Chen Y-C, Choe Y. Importance sampling and its optimality for stochastic simulation models. Electron J Statist. 2019;13(2):3386–423. doi: 10.1214/19-ejs1604

[9] Rubinstein RY. The cross-entropy method for combinatorial and continuous optimization. Methodol Comput Appl Prob. 1999;1(2):127–90.

[10] Akaike H. A new look at the statistical model identification. IEEE Trans Automat Contr. 1974;19(6):716–23. doi: 10.1109/tac.1974.1100705

[11] Donohue MC, Overholser R, Xu R, Vaida F. Conditional Akaike information under generalized linear and proportional hazards mixed models. Biometrika. 2011;98(3):685–700. doi: 10.1093/biomet/asr023 22822261 PMC3384357

[12] Claeskens G, Consentino F. Variable selection with incomplete covariate data. Biometrics. 2008;64(4):1062–9. doi: 10.1111/j.1541-0420.2008.01003.x 18371121

[13] Burnham KP, Anderson DR. Model selection and multimodel inference: a practical information-theoretic approach. New York: Springer; 2003.

[14] Claeskens G, Hjort NL. Model selection and model averaging. New York: Cambridge University Press; 2008.

[15] Bhansali RJ, Papangelou F. Convergence of moments of least squares estimators for the coefficients of an autoregressive process of unknown order. Ann Statist 1991;19(3):1155. doi: 10.1214/aos/1176348243

[16] Figueiredo MAT, Jain AK. Unsupervised learning of finite mixture models. IEEE Trans Pattern Anal Mach Intell. 2002;24(3):381–96. doi: 10.1109/34.990138

[17] Findley DF, Wei C-Z. AIC, overfitting principles, and the boundedness of moments of inverse matrices for vector autotregressions and related models. J Multivariate Anal. 2002;83(2):415–50. doi: 10.1006/jmva.2001.2063

[18] Bhansali RJ. A derivation of the information criteria for selecting autoregressive models. Adv Appl Prob. 1986;18(2):360–87. doi: 10.2307/1427304

[19] Hesterberg T. Weighted average importance sampling and defensive mixture distributions. Technometrics. 1995;37(2):185–94. doi: 10.1080/00401706.1995.10484303

[20] Owen A, Zhou Y. Safe and effective importance sampling. J Am Statist Assoc. 2000;95(449):135–43. doi: 10.1080/01621459.2000.10473909

[21] Cao QD, Choe Y. Cross-entropy based importance sampling for stochastic simulation models. Reliab Eng Syst Safety. 2019;191:106526. doi: 10.1016/j.ress.2019.106526

[22] Kurtz N, Song J. Cross-entropy-based adaptive importance sampling using Gaussian mixture. Struct Safety. 2013;42:35–44. doi: 10.1016/j.strusafe.2013.01.006

[23] Wang H, Zhou X. A cross-entropy scheme for mixtures. ACM Trans Model Comput Simul. 2015;25(1):1–20. doi: 10.1145/2685030

