# Supplementary material for: Information criterion for approximation of unnormalized densities
Source: PLoS One. 2025 Mar 17;20(3):e0317430. doi: 10.1371/journal.pone.0317430 (PMC11913297; doi:10.1371/journal.pone.0317430)
Supplement: S1 Appendices — It includes Appendix A (Assumptions and Proofs), Appendix B (EM Algorithm for Minimizing the Cross-Entropy), Appendix C (Numerical Experiments: Importance Sampling and Bayesian Inference), and Appendix D (Implementation Details of Numerical Experiments). (PDF) [file pone.0317430.s001.pdf]

## Appendices

This document includes Appendix A (Assumptions and Proofs), Appendix B (EM Algorithm for Minimizing the Cross-Entropy), Appendix C (Numerical Experiments: Importance Sampling and Bayesian Inference), and Appendix D (Implementation Details of Numerical Experiments), which refer to the equations in the main article.

## Appendix A: Assumptions and Proofs

We recall that the functions, $\mathcal{C}(\boldsymbol{\theta}) := -\mathbb{E}_\mu[r \log q_{\boldsymbol{\theta}}]$ and $h(\mathbf{X}, \boldsymbol{\eta}, \boldsymbol{\theta}) := \frac{r(\mathbf{X})}{q_{\boldsymbol{\eta}}(\mathbf{X})} \log q_{\boldsymbol{\theta}}(\mathbf{X})$, are defined in (5) and (S.1), respectively. Below, $\nabla_{\boldsymbol{\theta}}$ and $\nabla^2_{\boldsymbol{\theta}}$ denote the gradient and the Hessian with respect to $\boldsymbol{\theta}$, respectively. For example, $\nabla_{\boldsymbol{\theta}} h(\mathbf{X}, \boldsymbol{\eta}, \boldsymbol{\theta}^*)$ denotes the gradient of $h(\mathbf{X}, \boldsymbol{\eta}, \boldsymbol{\theta})$ with respect to $\boldsymbol{\theta}$ at $\boldsymbol{\theta}^*$.

**Assumptions**

(A) For $\boldsymbol{\theta}^* := \underset{\boldsymbol{\theta} \in \boldsymbol{\Theta}_d}{\operatorname{argmin}}\, \mathcal{C}(\boldsymbol{\theta})$,

(A1) the optimal distribution $Q^* = Q_{\boldsymbol{\theta}^*}$.

(A2) $\boldsymbol{\theta}^*$ is an interior point of $\boldsymbol{\Theta}_d$.

(A3) $\boldsymbol{\Gamma} := -\mathbb{E}_\mu[r\nabla^2_{\boldsymbol{\theta}} \log q_{\boldsymbol{\theta}^*}]$ is nonsingular.

(A4) $\nabla_{\boldsymbol{\theta}}(1/q_{\boldsymbol{\theta}}(\mathbf{x}))$ is continuous in a neighborhood (under the Euclidean norm) of $\boldsymbol{\theta}^*$ for a.e. $\mathbf{x}$ under $\mu$.

(A5) $\left\|\mathbb{E}_\mu\left[\nabla_{\boldsymbol{\theta}}(1/q_{\boldsymbol{\theta}})\,\nabla_{\boldsymbol{\theta}} q_{\boldsymbol{\theta}^*}\,(\nabla_{\boldsymbol{\theta}} q_{\boldsymbol{\theta}^*})^\top\right]\right\|$ is bounded for all $\boldsymbol{\theta} \in \boldsymbol{\Theta}_d$ and $\boldsymbol{\Theta}_d$ is a compact set.

(A6) There exists a measurable function $g_0(\mathbf{x})$ such that $\int g_0\,\mathrm{d}\mu < \infty$ and

$$\max\{\|\nabla_{\boldsymbol{\theta}} q_{\boldsymbol{\theta}}(\mathbf{x})\|, \|\nabla^2_{\boldsymbol{\theta}} q_{\boldsymbol{\theta}}(\mathbf{x})\|\} \le g_0(\mathbf{x})$$

for all $\boldsymbol{\theta} \in \boldsymbol{\Theta}_d$.

(B) For any $\boldsymbol{\eta} \in \boldsymbol{\Theta}_d$,

(B1) $h(\mathbf{x}, \boldsymbol{\eta}, \cdot)$ is continuous on $\boldsymbol{\Theta}_d$ for a.e. $\mathbf{x}$ under $Q_{\boldsymbol{\eta}}$.

(B2) there exists a measurable function $g_{\boldsymbol{\eta}}$ such that $\int |g_{\boldsymbol{\eta}}|\,\mathrm{d}Q_{\boldsymbol{\eta}} < \infty$ and $|h(\mathbf{x}, \boldsymbol{\eta}, \boldsymbol{\theta})| \le g_{\boldsymbol{\eta}}(\mathbf{x})$ for a.e. $\mathbf{x}$ under $Q_{\boldsymbol{\eta}}$ and all $\boldsymbol{\theta} \in \boldsymbol{\Theta}_d$.

(B3) $h(\mathbf{x}, \boldsymbol{\eta}, \cdot)$ is thrice continuously differentiable on $\boldsymbol{\Theta}_d$ for a.e. $\mathbf{x}$ under $Q_{\boldsymbol{\eta}}$.

(B4) there exist measurable functions $g^{(i)}_{\boldsymbol{\eta}}$ such that $\int |g^{(i)}_{\boldsymbol{\eta}}|^4\,\mathrm{d}Q_{\boldsymbol{\eta}} < \infty$ and $\|\nabla^i_{\boldsymbol{\theta}} h(\mathbf{x}, \boldsymbol{\eta}, \boldsymbol{\theta})\|_{\max} \le g^{(i)}_{\boldsymbol{\eta}}(\mathbf{x})$ for $i = 1, 2, 3$ and all $\boldsymbol{\theta} \in \boldsymbol{\Theta}_d$. The norm is the elementwise maximal norm.

(B5) $\boldsymbol{\Lambda}_{\boldsymbol{\eta}} := \mathbb{E}_{Q_{\boldsymbol{\eta}}}\left[\nabla_{\boldsymbol{\theta}} h(\mathbf{X}, \boldsymbol{\eta}, \boldsymbol{\theta}^*) \nabla_{\boldsymbol{\theta}} h(\mathbf{X}, \boldsymbol{\eta}, \boldsymbol{\theta}^*)^\top\right]$ exists and every element of $\nabla^2_{\boldsymbol{\theta}} h(\mathbf{X}, \boldsymbol{\eta}, \boldsymbol{\theta}^*)$ is a sub-Gaussian random variable where $\mathbf{X} \sim Q_{\boldsymbol{\eta}}$.

Assumptions (A1-3) and (B1-4) are the standard regularity conditions to establish consistency (Lemma 1) and asymptotic normality (Lemma 2) of an M-estimator. Assumptions (B3) and (B4) imply Assumptions (B1) and (B2), respectively, as Lemma 2 requires stronger assumptions than Lemma 1.

To establish Lemma 3 and Theorem 1, additional assumptions (A4-6) and (B5) are needed because we need to control the expectation of the smaller order terms in the asymptotic analysis. Assumptions (A4-6) ensure that we can exchange the expectation and derivative of the function $h(\mathbf{X}, \boldsymbol{\eta}, \boldsymbol{\theta})$. Assumption (B5) ensures that every moment of every element in the Hessian matrix exists. This will be needed when deriving the convergence of sample Hessian matrix to the population Hessian matrix.

A crucial difference from the conventional analysis on the asymptotic normality is that to derive the AIC and CIC, we need expectation bound on the smaller order terms. The asymptotic normality only requires convergence in probability and distribution, which are not enough for the AIC and CIC. Also, the bounded derivatives up to the third (not second) order

in (B4) is an additional requirement that allows us to bound the Taylor's remainder term. This is needed because there is no multivariate mean value theorem.

Note that assumptions (B4-5) imply the uniform integrability conditions that are commonly used in the literature for information criteria akin to the AIC to make the model complexity penalty to be expressed in the free parameter dimension $d$ (see Conditions A7–A8 in Donohue et al. [2011], Theorem 1 in Claeskens and Consentino [2008], and more references cited in Bhansali and Papangelou [1991, p.1157] and Findley and Wei [2002, p.416]). Essentially, the uniform integrability condition is to make sure the expectation of the smaller order terms is still a smaller order term. Here we use bounds on derivatives and Taylor remainder theorem to handle this.

We first establish the properties of the MCE (Lemmas 1–3) before Theorem 1. The MCE is a minimizer of (6) so it is an M-estimator, which allows us to use the M-estimation theory [Van der Vaart, 1998]. The MCE $\widehat{\boldsymbol{\theta}}_n$ is a strongly consistent estimator of $\boldsymbol{\theta}^* := \underset{\boldsymbol{\theta}\in\boldsymbol{\Theta}_d}{\operatorname{argmin}}\, \mathcal{C}(\boldsymbol{\theta})$ (Lemma 1) and has asymptotic normality (Lemma 2) under standard regularity conditions.

**Lemma 1** (Strong consistency of the MCE)**.** *Suppose that assumptions (A1) and (B1-2) hold and* $\boldsymbol{\Theta}_d$ *is compact. Then, for any* $\boldsymbol{\eta} \in \boldsymbol{\Theta}_d$*, the MCE* $\widehat{\boldsymbol{\theta}}_n := \underset{\boldsymbol{\theta}\in\boldsymbol{\Theta}_d}{\operatorname{argmin}}\, \bar{\mathcal{C}}_{\boldsymbol{\eta}}(\boldsymbol{\theta})$ *converges almost surely to* $\boldsymbol{\theta}^*$ *as* $n \to \infty$.

**Lemma 2** (Asymptotic normality of the MCE). *Suppose that assumptions (A1-3) and (B3-4) hold and that for any* $\boldsymbol{\eta} \in \boldsymbol{\Theta}_d$, $\widehat{\boldsymbol{\theta}}_n$ *converges in probability to* $\boldsymbol{\theta}^*$ *as* $n \to \infty$. *Then, for any* $\boldsymbol{\eta} \in \boldsymbol{\Theta}_d$, $\sqrt{n}\left(\widehat{\boldsymbol{\theta}}_n - \boldsymbol{\theta}^*\right)$ *converges in distribution to* $\mathcal{N}\big(0, \boldsymbol{\Gamma}^{-1}\boldsymbol{\Lambda}_{\boldsymbol{\eta}}\boldsymbol{\Gamma}^{-1}\big)$ *as* $n \to \infty$, *where* $\boldsymbol{\Gamma} := -\mathbb{E}_\mu[r\nabla^2_{\boldsymbol{\theta}} \log q_{\boldsymbol{\theta}^*}]$ *and* $\boldsymbol{\Lambda}_{\boldsymbol{\eta}} := \mathbb{E}_{Q_{\boldsymbol{\eta}}}\big[\nabla_{\boldsymbol{\theta}} h(\mathbf{X}, \boldsymbol{\eta}, \boldsymbol{\theta}^*)\nabla_{\boldsymbol{\theta}} h(\mathbf{X}, \boldsymbol{\eta}, \boldsymbol{\theta}^*)^\top\big]$ *with*

$$h(\mathbf{X}, \boldsymbol{\eta}, \boldsymbol{\theta}) := \frac{r(\mathbf{X})}{q_{\boldsymbol{\eta}}(\mathbf{X})} \log q_{\boldsymbol{\theta}}(\mathbf{X}). \tag{S.1}$$

The above two lemmas are common results from the standard assumptions, see, e.g., Theorem A1 and A2 in Rubinstein and Shapiro [1993]. The next lemma characterizes the limiting behavior of any MCE.

**Lemma 3.** *Suppose that assumptions (A1-5) and (B1-5) hold and let* $\widehat{\boldsymbol{\theta}}_n$ *be the MCE. Then* $\sqrt{n}\left(\widehat{\boldsymbol{\theta}}_n - \boldsymbol{\theta}^*\right) = Z_{\boldsymbol{\eta}} + \epsilon_{n,\boldsymbol{\eta}}$ *where*

$$Z_{\boldsymbol{\eta}} := -\boldsymbol{\Gamma}^{-1}\frac{1}{\sqrt{n}}\sum_{i=1}^{n} \nabla_{\boldsymbol{\theta}} h(\mathbf{X}_i; \boldsymbol{\eta}, \boldsymbol{\theta}^*), \quad \sup_{\boldsymbol{\eta}\in\boldsymbol{\Theta}_d} \mathbb{E}(\|\epsilon_{n,\boldsymbol{\eta}}\|) = O(1/n^{1/4})$$

*and* $\mathbb{E}(Z_{\boldsymbol{\eta}}) = 0, \quad \mathsf{Cov}(Z_{\boldsymbol{\eta}}) = \boldsymbol{\Gamma}^{-1}\boldsymbol{\Lambda}_{\boldsymbol{\eta}}\boldsymbol{\Gamma}^{-1}, \quad \boldsymbol{\Lambda}_{\boldsymbol{\eta}} = \rho\boldsymbol{\Gamma} + O(\|\boldsymbol{\eta} - \boldsymbol{\theta}^*\|).$

Lemma 3 is different from the conventional asymptotic normality (e.g., Lemma 2) in two senses. First, we require a bound on the expectation of the remainder term $\mathbb{E}(\|\epsilon_{n,\boldsymbol{\eta}}\|)$, which implies the rate of $O_P(1/n^{1/4})$ and the asymptotic normality. We need the expectation because the derivation

of the asymptotic bias in the CIC analysis requires an expectation bound. Second, the expectation of the remainder term is bounded uniformly for all $\boldsymbol{\eta} \in \boldsymbol{\Theta}_d$. This is in contrast to the conventional analysis that only requires the result at $\boldsymbol{\eta} = \boldsymbol{\theta}^*$. We need the uniform bound over $\boldsymbol{\eta} \in \boldsymbol{\Theta}_d$ because the data are generated from a measure $Q_{\boldsymbol{\eta}}$ where $\boldsymbol{\eta}$ is a random vector. In our analysis, we generate a sequence of estimators $\widehat{\boldsymbol{\theta}}_n^{(1)}, \widehat{\boldsymbol{\theta}}_n^{(2)}, \cdots, \widehat{\boldsymbol{\theta}}_n^{(\tau)}$ and the data used to estimate $\widehat{\boldsymbol{\theta}}_n^{(t)}$ are generated from $Q_{\widehat{\boldsymbol{\theta}}_n^{(t-1)}}$, the measure based on the previous set of data. The uniform bound ensures that the remainder term $\epsilon_{n,\boldsymbol{\eta}}$ is small even if $\boldsymbol{\eta}$ is a random quantity. For any sequential/iterative sampling procedure, we would need a similar bound to control the remainder terms.

Below, we provide the proofs of Lemma 3 and Theorem 1.

*Proof of Lemma 3.* We define the directional derivative with respect to variable $\boldsymbol{\theta} \in \boldsymbol{\Theta}_d \subset \mathbb{R}^d$ in the direction $v \in \mathbb{R}^d$ as

$$\nabla_{\boldsymbol{\theta},v} := v^\top \nabla_{\boldsymbol{\theta}}.$$

Recall that $\boldsymbol{\theta}^* := \operatorname*{argmin}_{\boldsymbol{\theta}\in\boldsymbol{\Theta}_d} \mathcal{C}(\boldsymbol{\theta})$ is the population minimizer and

$$\begin{aligned}\widehat{\boldsymbol{\theta}}_n &= \operatorname*{argmin}_{\boldsymbol{\theta}\in\boldsymbol{\Theta}_d} \bar{\mathcal{C}}_{\boldsymbol{\eta}}(\boldsymbol{\theta}) \\ &= \operatorname*{argmin}_{\boldsymbol{\theta}\in\boldsymbol{\Theta}_d} \frac{1}{n}\sum_{i=1}^{n} h(\mathbf{X}_i;\boldsymbol{\eta},\boldsymbol{\theta}) \\ &= \operatorname*{argmin}_{\boldsymbol{\theta}\in\boldsymbol{\Theta}_d} h_n(\boldsymbol{\eta},\boldsymbol{\theta})\end{aligned}$$

is the estimator, where $\mathbf{X}_i \sim Q_{\boldsymbol{\eta}}$ and

$$h_n(\boldsymbol{\eta},\boldsymbol{\theta}) := \frac{1}{n}\sum_{i=1}^{n} h(\mathbf{X}_i;\boldsymbol{\eta},\boldsymbol{\theta}).$$

The expectation of $h_n$ is

$$\mathbb{E}_{Q_{\boldsymbol{\eta}}}(h(\mathbf{X}_i;\boldsymbol{\eta},\boldsymbol{\theta})) = \mathcal{C}(\boldsymbol{\theta})$$

for all $\boldsymbol{\eta} \in \boldsymbol{\Theta}_d$.

Because $\boldsymbol{\theta}^*$ and $\widehat{\boldsymbol{\theta}}_n$ are the minimizers, they solve the score equations

$$0 = \nabla_{\boldsymbol{\theta}}\mathcal{C}(\boldsymbol{\theta}^*) = \nabla_{\boldsymbol{\theta}} h_n(\boldsymbol{\eta},\widehat{\boldsymbol{\theta}}_n).$$

Thus, by the Taylor's remainder theorem,

$$
\begin{aligned}
-\nabla_{\boldsymbol{\theta}} h_n(\boldsymbol{\eta}, \boldsymbol{\theta}^*) &= \nabla_{\boldsymbol{\theta}} h_n(\boldsymbol{\eta}, \widehat{\boldsymbol{\theta}}_n) - \nabla_{\boldsymbol{\theta}} h_n(\boldsymbol{\eta}, \boldsymbol{\theta}^*) \\
&= \nabla_{\boldsymbol{\theta}, \widehat{\boldsymbol{\theta}}_n - \boldsymbol{\theta}^*} \nabla_{\boldsymbol{\theta}} h_n(\boldsymbol{\eta}, \boldsymbol{\theta}^*) + \int_{\epsilon=0}^{\epsilon=1} \nabla_{\boldsymbol{\theta}, \widehat{\boldsymbol{\theta}}_n - \boldsymbol{\theta}^*} \nabla_{\boldsymbol{\theta}, \widehat{\boldsymbol{\theta}}_n - \boldsymbol{\theta}^*} \nabla_{\boldsymbol{\theta}} h_n(\boldsymbol{\eta}, \boldsymbol{\theta}^* + \epsilon(\widehat{\boldsymbol{\theta}}_n - \boldsymbol{\theta}^*))\, \mathrm{d}\epsilon \\
&= \boldsymbol{\Gamma}_n(\boldsymbol{\eta}, \boldsymbol{\theta}^*)(\widehat{\boldsymbol{\theta}}_n - \boldsymbol{\theta}^*) + R_{1,n}(\boldsymbol{\eta}),
\end{aligned}
$$

where

$$
\begin{aligned}
\boldsymbol{\Gamma}_n(\boldsymbol{\eta}, \boldsymbol{\theta}^*) &:= \nabla^2_{\boldsymbol{\theta}} h_n(\boldsymbol{\eta}, \boldsymbol{\theta}^*), \\
R_{1,n}(\boldsymbol{\eta}) &:= \int_{\epsilon=0}^{\epsilon=1} \nabla_{\boldsymbol{\theta}, \widehat{\boldsymbol{\theta}}_n - \boldsymbol{\theta}^*} \nabla_{\boldsymbol{\theta}, \widehat{\boldsymbol{\theta}}_n - \boldsymbol{\theta}^*} \nabla_{\boldsymbol{\theta}} h_n(\boldsymbol{\eta}, \boldsymbol{\theta}^* + \epsilon(\widehat{\boldsymbol{\theta}}_n - \boldsymbol{\theta}^*))\, \mathrm{d}\epsilon.
\end{aligned}
$$

$R_{1,n}(\boldsymbol{\eta})$ represents the remainder terms.

Thus, when $\boldsymbol{\Gamma}_n(\boldsymbol{\eta}, \boldsymbol{\theta}^*)$ is invertible, the difference in the estimator can be expressed as

$$
\sqrt{n}(\widehat{\boldsymbol{\theta}}_n - \boldsymbol{\theta}^*) = -\sqrt{n}\boldsymbol{\Gamma}_n^{-1}(\boldsymbol{\eta}, \boldsymbol{\theta}^*)\nabla_{\boldsymbol{\theta}} h_n(\boldsymbol{\eta}, \boldsymbol{\theta}^*) - \sqrt{n}\boldsymbol{\Gamma}_n^{-1}(\boldsymbol{\eta}, \boldsymbol{\theta}^*) R_{1,n}(\boldsymbol{\eta}). \quad \text{(S.2)}
$$

The first quantity is how we establish the asymptotic normality and the second part (remainder term) is something we need to bound its expectation.

**Bounding the remainder term $R_{1,n}(\boldsymbol{\eta})$.** To see how we obtain this,

first note that

$$\mathbb{E}(\|\boldsymbol{\Gamma}_n^{-1}(\boldsymbol{\eta},\boldsymbol{\theta}^*)R_{1,n}(\boldsymbol{\eta})\|) \leq \sqrt{\mathbb{E}(\|\boldsymbol{\Gamma}_n^{-1}(\boldsymbol{\eta},\boldsymbol{\theta}^*)\|^2)\mathbb{E}(\|R_{1,n}(\boldsymbol{\eta})\|^2)}. \qquad \text{(S.3)}$$

Here both norms are the 2-norm for matrices and vectors. So it suffices to bound the two terms individually.

**Bounding $\mathbb{E}(\|\boldsymbol{\Gamma}_n^{-1}(\boldsymbol{\eta},\boldsymbol{\theta}^*)\|^2)$.** To bound the inverse matrix part, recall that $\boldsymbol{\Gamma} = \nabla^2_{\boldsymbol{\theta}}\mathcal{C}(\boldsymbol{\theta}^*)$ and note that since $\mathbb{E}(\boldsymbol{\Gamma}_n(\boldsymbol{\eta},\boldsymbol{\theta}^*)) = \boldsymbol{\Gamma}$ due to assumption (A6) and $\boldsymbol{\Gamma}_n(\boldsymbol{\eta},\boldsymbol{\theta}^*)$ is essentially a sample mean matrix so the variance shrinks at rate $O(1/n)$ due to assumption (B5). Thus, we can decompose

$$\boldsymbol{\Gamma}_n(\boldsymbol{\eta},\boldsymbol{\theta}^*) = \boldsymbol{\Gamma} + \Delta_{n,\boldsymbol{\Gamma}}(\boldsymbol{\eta}) \qquad \text{(S.4)}$$

with $\mathbb{E}(\|\Delta_{n,\boldsymbol{\Gamma}}(\boldsymbol{\eta})\|) = O(1/\sqrt{n})$. Moreover, because assumption (B5) holds for all $\boldsymbol{\eta} \in \boldsymbol{\Theta}_d$,

$$\sup_{\boldsymbol{\eta}\in\boldsymbol{\Theta}_d} \mathbb{E}(\|\Delta_{n,\boldsymbol{\Gamma}}(\boldsymbol{\eta})\|) = O(1/\sqrt{n}).$$

For the inverse matrix, because $(I+A)^{-1} = I - A + A^2 - A^3 + \cdots$ and the fact that $\Delta_{n,\boldsymbol{\Gamma}}(\boldsymbol{\eta})$ is sub-Gaussian due to (B5), we have that

$$\mathbb{E}\|\Delta_{n,\boldsymbol{\Gamma}}(\boldsymbol{\eta})\|^p = O\left(n^{-p/2}\right) \qquad \text{(S.5)}$$

for any $p > 2$ and that

$$
\begin{aligned}
\boldsymbol{\Gamma}_n^{-1}(\boldsymbol{\eta}, \boldsymbol{\theta}^*) &= [\boldsymbol{\Gamma} + \Delta_{n,\boldsymbol{\Gamma}}(\boldsymbol{\eta})]^{-1} \\
&= [\boldsymbol{\Gamma}(I + \boldsymbol{\Gamma}^{-1}\Delta_{n,\boldsymbol{\Gamma}}(\boldsymbol{\eta}))]^{-1} \\
&= (I - \boldsymbol{\Gamma}^{-1}\Delta_{n,\boldsymbol{\Gamma}}(\boldsymbol{\eta}) + O_P(\|\Delta_{n,\boldsymbol{\Gamma}}(\boldsymbol{\eta})\|^2))\boldsymbol{\Gamma}^{-1} \\
&= \boldsymbol{\Gamma}^{-1} - \boldsymbol{\Gamma}^{-1}\Delta_{n,\boldsymbol{\Gamma}}(\boldsymbol{\eta})\boldsymbol{\Gamma}^{-1} + O_P(\|\Delta_{n,\boldsymbol{\Gamma}}(\boldsymbol{\eta})\|^2).
\end{aligned}
$$

Note that the higher order terms in the above expansion can be ignored due to equation (S.5).

By the fact that $\boldsymbol{\Gamma}$ is invertible and $\sup_{\boldsymbol{\eta}\in\boldsymbol{\Theta}_d} \mathbb{E}(\|\Delta_{n,\boldsymbol{\Gamma}}(\boldsymbol{\eta})\|) = O(1/\sqrt{n})$, we conclude that

$$
\sup_{\boldsymbol{\eta}\in\boldsymbol{\Theta}_d} \mathbb{E}(\|\boldsymbol{\Gamma}_n^{-1}(\boldsymbol{\eta}, \boldsymbol{\theta}^*)\|) = \|\boldsymbol{\Gamma}^{-1}\| + O(1/\sqrt{n}) \tag{S.6}
$$

and

$$
\sup_{\boldsymbol{\eta}\in\boldsymbol{\Theta}_d} \mathbb{E}(\|\boldsymbol{\Gamma}_n^{-1}(\boldsymbol{\eta}, \boldsymbol{\theta}^*)\|^2) = \|\boldsymbol{\Gamma}^{-1}\|^2 + O(1/\sqrt{n}). \tag{S.7}
$$

**Bounding $\mathbb{E}(\|R_{1,n}(\boldsymbol{\eta})\|^2)$.** By the definition of $R_{1,n}(\boldsymbol{\eta})$,

$$
R_{1,n}(\boldsymbol{\eta}) = \int_{\epsilon=0}^{\epsilon=1} \nabla_{\boldsymbol{\theta},\widehat{\boldsymbol{\theta}}_n-\boldsymbol{\theta}^*} \nabla_{\boldsymbol{\theta},\widehat{\boldsymbol{\theta}}_n-\boldsymbol{\theta}^*} \nabla_{\boldsymbol{\theta}} h_n(\boldsymbol{\eta}, \boldsymbol{\theta}^* + \epsilon(\widehat{\boldsymbol{\theta}}_n - \boldsymbol{\theta}^*))\, \mathrm{d}\epsilon
$$

so its norm can be bounded by

$$
\begin{aligned}
\|R_{1,n}(\boldsymbol{\eta})\| &\leq \|\widehat{\boldsymbol{\theta}}_n - \boldsymbol{\theta}^*\|^2 \sup_{\boldsymbol{\theta}\in\boldsymbol{\Theta}_d} \sup_{\|v\|=\|u\|=\|w\|=1} \|\nabla_{\boldsymbol{\theta},v}\nabla_{\boldsymbol{\theta},u}\nabla_{\boldsymbol{\theta},w} h_n(\boldsymbol{\eta},\boldsymbol{\theta})\| \\
&\leq \|\widehat{\boldsymbol{\theta}}_n - \boldsymbol{\theta}^*\|^2 d^{3/2} \int g^{(3)}_{\boldsymbol{\eta}} \, \mathrm{d}\widehat{Q}_{\boldsymbol{\eta}}
\end{aligned}
$$

using assumption (B4) and $d$ is the dimension of $\boldsymbol{\theta}$ and $\widehat{Q}_{\boldsymbol{\eta}}$ is the empirical measure formed by $\mathbf{X}_1, \cdots, \mathbf{X}_n$.

Because assumption (B4) holds for all $\boldsymbol{\theta}$ and $\boldsymbol{\eta}$,

$$
\begin{aligned}
\sup_{\boldsymbol{\eta}\in\boldsymbol{\Theta}_d} \mathbb{E}\|R_{1,n}(\boldsymbol{\eta})\|^2 &\leq \sup_{\boldsymbol{\eta}\in\boldsymbol{\Theta}_d} d^3 \mathbb{E}\left(\|\widehat{\boldsymbol{\theta}}_n - \boldsymbol{\theta}^*\|^4 \left(\int g^{(3)}_{\boldsymbol{\eta}} \, \mathrm{d}\widehat{Q}_{\boldsymbol{\eta}}\right)^2\right) \\
&\leq d^3 \sqrt{\mathbb{E}\left(\|\widehat{\boldsymbol{\theta}}_n - \boldsymbol{\theta}^*\|^8\right) \sup_{\boldsymbol{\eta}\in\boldsymbol{\Theta}_d} \mathbb{E}\left(\left(\int g^{(3)}_{\boldsymbol{\eta}} \, \mathrm{d}\widehat{Q}_{\boldsymbol{\eta}}\right)^4\right)} \qquad \text{(S.8)} \\
&= O\left(\sqrt{\mathbb{E}\|\widehat{\boldsymbol{\theta}}_n - \boldsymbol{\theta}^*\|^8}\right) = O(1/n^2).
\end{aligned}
$$

Putting it altogether, we obtain

$$
\begin{aligned}
\sup_{\boldsymbol{\eta}\in\boldsymbol{\Theta}_d} \sqrt{n}\mathbb{E}(\|\boldsymbol{\Gamma}_n^{-1}(\boldsymbol{\eta},\boldsymbol{\theta}^*)R_{1,n}(\boldsymbol{\eta})\|) &\leq \sup_{\boldsymbol{\eta}\in\boldsymbol{\Theta}_d} \sqrt{n}\sqrt{\mathbb{E}(\|\boldsymbol{\Gamma}_n^{-1}(\boldsymbol{\eta},\boldsymbol{\theta}^*)\|^2)\mathbb{E}(\|R_{1,n}(\boldsymbol{\eta})\|^2)} \\
&= \sqrt{n}(\|\boldsymbol{\Gamma}^{-1}\| + O(1/n^{1/4})) \cdot O\left(\sqrt{\mathbb{E}\|\widehat{\boldsymbol{\theta}}_n - \boldsymbol{\theta}^*\|^4}\right) \\
&= O(1/\sqrt{n}) = o(1).
\end{aligned}
$$

**Analysis of the first order term in equation** (S.2)**.** Note that the

difference between the first order term and $Z_{\boldsymbol{\eta}}$ is

$$R_{2,n}(\boldsymbol{\eta}) := \sqrt{n}\boldsymbol{\Gamma}_n^{-1}(\boldsymbol{\eta},\boldsymbol{\theta}^*)\nabla_{\boldsymbol{\theta}} h_n(\boldsymbol{\eta},\boldsymbol{\theta}^*) - Z_{\boldsymbol{\eta}} = \sqrt{n}(\boldsymbol{\Gamma}_n^{-1}(\boldsymbol{\eta},\boldsymbol{\theta}^*) - \boldsymbol{\Gamma}^{-1})\nabla_{\boldsymbol{\theta}} h_n(\boldsymbol{\eta},\boldsymbol{\theta}^*)$$

so we need to show that

$$\sup_{\boldsymbol{\eta}\in\boldsymbol{\Theta}_d} \mathbb{E}\|R_{2,n}(\boldsymbol{\eta})\| = o(1).$$

We can decompose the above bound via

$$\sup_{\boldsymbol{\eta}\in\boldsymbol{\Theta}_d} \mathbb{E}\|R_{2,n}(\boldsymbol{\eta})\| \le \sup_{\boldsymbol{\eta}\in\boldsymbol{\Theta}_d} \sqrt{\mathbb{E}\left\|\boldsymbol{\Gamma}_n^{-1}(\boldsymbol{\eta},\boldsymbol{\theta}^*) - \boldsymbol{\Gamma}^{-1}\right\|^2 \cdot n\mathbb{E}\left\|\nabla_{\boldsymbol{\theta}} h_n(\boldsymbol{\eta},\boldsymbol{\theta}^*)\right\|^2}.$$

Using the derivation of equation (S.6), we can easily show that

$$\sup_{\boldsymbol{\eta}\in\boldsymbol{\Theta}_d} \mathbb{E}\|\boldsymbol{\Gamma}_n^{-1}(\boldsymbol{\eta},\boldsymbol{\theta}^*) - \boldsymbol{\Gamma}^{-1}\|^2 = O(1/\sqrt{n})$$

and the second part

$$n\mathbb{E}\|\nabla_{\boldsymbol{\theta}} h_n(\boldsymbol{\eta},\boldsymbol{\theta}^*)\|^2 = O(1)$$

because the covariance matrix $\mathsf{Cov}(\nabla_{\boldsymbol{\theta}} h_n(\boldsymbol{\eta},\boldsymbol{\theta}^*)) = \frac{1}{n}\boldsymbol{\Lambda}_{\boldsymbol{\eta}}$, where

$$\boldsymbol{\Lambda}_{\boldsymbol{\eta}} = \mathbb{E}_{Q_{\boldsymbol{\eta}}}\left[\nabla_{\boldsymbol{\theta}} h\left(\mathbf{X},\boldsymbol{\eta},\boldsymbol{\theta}^*\right)\nabla_{\boldsymbol{\theta}} h\left(\mathbf{X},\boldsymbol{\eta},\boldsymbol{\theta}^*\right)^\top\right]$$

is a bounded matrix for all $\boldsymbol{\eta}$ due to assumption (B5) and $\mathbb{E}\|\nabla_{\boldsymbol{\theta}} h_n(\boldsymbol{\eta}, \boldsymbol{\theta}^*)\|^2 = \mathsf{Tr}(\mathsf{Cov}(\nabla_{\boldsymbol{\theta}} h_n(\boldsymbol{\eta}, \boldsymbol{\theta}^*)))$. $\mathsf{Tr}(\boldsymbol{A})$ is the trace of the matrix $\boldsymbol{A}$.

Equation (S.2) also implies a useful result about the limiting behavior of $\sqrt{n}(\widehat{\boldsymbol{\theta}}_n - \boldsymbol{\theta}^*)$. First, because $\boldsymbol{\Gamma}_n^{-1}(\boldsymbol{\eta}, \boldsymbol{\theta}^*)$ converges in probability to $\boldsymbol{\Gamma}^{-1}$ and $\nabla_{\boldsymbol{\theta}} h_n(\boldsymbol{\eta}, \boldsymbol{\theta}^*)$ is a sample average quantity, we have a central limit theorem about $\sqrt{n}(\widehat{\boldsymbol{\theta}}_n - \boldsymbol{\theta}^*)$ in the sense that

$$\sqrt{n}(\widehat{\boldsymbol{\theta}}_n - \boldsymbol{\theta}^*) \xrightarrow{D} \mathcal{N}(0, \boldsymbol{\Sigma}_{\boldsymbol{\eta}}),$$

where $\boldsymbol{\Sigma}_{\boldsymbol{\eta}} := \boldsymbol{\Gamma}^{-1}\boldsymbol{\Lambda}_{\boldsymbol{\eta}}\boldsymbol{\Gamma}^{-1}$ and $\boldsymbol{\Sigma}_{\boldsymbol{\eta}}$ is the asymptotic variance of $\widehat{\boldsymbol{\theta}}_n - \boldsymbol{\theta}^*$, i.e.,

$$n\mathsf{Var}(\widehat{\boldsymbol{\theta}}_n - \boldsymbol{\theta}^*) \to \boldsymbol{\Sigma}_{\boldsymbol{\eta}}.$$

Putting it altogether, we conclude that

$$\sup_{\boldsymbol{\eta} \in \boldsymbol{\Theta}_d} \mathbb{E}\|R_{2,n}(\boldsymbol{\eta})\| = O(1/n^{1/4})$$

and we can rewrite equation (S.2) as

$$\begin{aligned}\sqrt{n}(\widehat{\boldsymbol{\theta}}_n - \boldsymbol{\theta}^*) &= -\sqrt{n}\boldsymbol{\Gamma}_n^{-1}(\boldsymbol{\eta}, \boldsymbol{\theta}^*)\nabla_{\boldsymbol{\theta}} h_n(\boldsymbol{\eta}, \boldsymbol{\theta}^*) - \sqrt{n}\boldsymbol{\Gamma}_n^{-1}(\boldsymbol{\eta}, \boldsymbol{\theta}^*)R_{1,n}(\boldsymbol{\eta}) \\ &= -Z_{\boldsymbol{\eta}} \underbrace{-\sqrt{n}\boldsymbol{\Gamma}_n^{-1}(\boldsymbol{\eta}, \boldsymbol{\theta}^*)R_{1,n}(\boldsymbol{\eta}) - R_{2,n}(\boldsymbol{\eta})}_{:=\epsilon_{n,\boldsymbol{\eta}}}\end{aligned}$$

with $\sup_{\boldsymbol{\eta}\in\boldsymbol{\Theta}_d} \mathbb{E}(\|\epsilon_{n,\boldsymbol{\eta}}\|) = O(1/n^{1/4})$.

The covariance of $Z_{\boldsymbol{\eta}}$ is

$$\mathsf{Cov}(Z_{\boldsymbol{\eta}}) = \boldsymbol{\Gamma}^{-1}\boldsymbol{\Lambda}_{\boldsymbol{\eta}}\boldsymbol{\Gamma}^{-1}$$

and we have

$$\begin{aligned}
\boldsymbol{\Lambda}_{\boldsymbol{\eta}} &= \mathbb{E}_{Q_{\boldsymbol{\eta}}}\left[\nabla_{\boldsymbol{\theta}} h\left(\mathbf{X},\boldsymbol{\eta},\boldsymbol{\theta}^*\right)\nabla_{\boldsymbol{\theta}} h\left(\mathbf{X},\boldsymbol{\eta},\boldsymbol{\theta}^*\right)^\top\right] \\
&= \mathbb{E}_{Q_{\boldsymbol{\eta}}}\left[\frac{r^2(\mathbf{X})}{q^2_{\boldsymbol{\eta}}(\mathbf{X})}\nabla_{\boldsymbol{\theta}}\log q_{\boldsymbol{\theta}^*}(\mathbf{X})\left(\nabla_{\boldsymbol{\theta}}\log q_{\boldsymbol{\theta}^*}(\mathbf{X})\right)^\top\right] \\
&= \mathbb{E}_{\mu}\left[\frac{r^2}{q_{\boldsymbol{\eta}}}\nabla_{\boldsymbol{\theta}}\log q_{\boldsymbol{\theta}^*}\left(\nabla_{\boldsymbol{\theta}}\log q_{\boldsymbol{\theta}^*}\right)^\top\right] \\
&= \mathbb{E}_{\mu}\left[\frac{r^2}{q_{\boldsymbol{\eta}}q^2_{\boldsymbol{\theta}^*}}\nabla_{\boldsymbol{\theta}} q_{\boldsymbol{\theta}^*}\left(\nabla_{\boldsymbol{\theta}} q_{\boldsymbol{\theta}^*}\right)^\top\right] \\
&= \rho^2\mathbb{E}_{\mu}\left[\frac{1}{q_{\boldsymbol{\eta}}}\nabla_{\boldsymbol{\theta}} q_{\boldsymbol{\theta}^*}\left(\nabla_{\boldsymbol{\theta}} q_{\boldsymbol{\theta}^*}\right)^\top\right] && \text{(S.9)} \\
&= \rho^2\int\left(\frac{1}{q_{\boldsymbol{\theta}^*}(\mathbf{x})} + R_{3,n}(\mathbf{x};\boldsymbol{\eta})\right)\nabla_{\boldsymbol{\theta}} q_{\boldsymbol{\theta}^*}(\mathbf{x})\left(\nabla_{\boldsymbol{\theta}} q_{\boldsymbol{\theta}^*}(\mathbf{x})\right)^\top \mathrm{d}\mu(\mathbf{x}) && \text{(S.10)} \\
&= \rho\boldsymbol{\Gamma} + \rho^2\int R_{3,n}(\mathbf{x};\boldsymbol{\eta})\nabla_{\boldsymbol{\theta}} q_{\boldsymbol{\theta}^*}(\mathbf{x})\left(\nabla_{\boldsymbol{\theta}} q_{\boldsymbol{\theta}^*}(\mathbf{x})\right)^\top \mathrm{d}\mu(\mathbf{x}) \\
&\overset{(A5)}{=} \rho\boldsymbol{\Gamma} + O(\|\boldsymbol{\eta}-\boldsymbol{\theta}^*\|), && \text{(S.11)}
\end{aligned}$$

where

$$R_{3,n}(\mathbf{x};\boldsymbol{\eta}) := \int_{\epsilon=0}^{\epsilon=1}\left(\boldsymbol{\eta}-\boldsymbol{\theta}^*\right)^\top\nabla_{\boldsymbol{\theta}}\left(\frac{1}{q_{\boldsymbol{\theta}^*+\epsilon(\boldsymbol{\eta}-\boldsymbol{\theta}^*)}(\mathbf{x})}\right)\mathrm{d}\epsilon$$

is the Taylor's remainder term.

Therefore,

$$n\mathsf{Var}(\widehat{\boldsymbol{\theta}}_n - \boldsymbol{\theta}^*) = \boldsymbol{\Sigma}_{\boldsymbol{\eta}} + o(1) = \rho\boldsymbol{\Gamma}^{-1} + o(1) + O(\|\boldsymbol{\eta} - \boldsymbol{\theta}^*\|).$$

$\square$

*Proof of Theorem 1.* We first simplify $\mathcal{C}\left(\widehat{\boldsymbol{\theta}}_n^{(t)}\right)$ and $\bar{\mathcal{C}}_{\widehat{\boldsymbol{\theta}}_n^{(t-1)}}\left(\widehat{\boldsymbol{\theta}}_n^{(t)}\right)$. We then use the simplified expressions to derive the bias of interest. The first quantity is a population objective function while the second quantity is a sample objective function.

**Analysis on the population objective function $\mathcal{C}\left(\widehat{\boldsymbol{\theta}}_n^{(t)}\right)$.**

$$\begin{aligned}\mathcal{C}\left(\widehat{\boldsymbol{\theta}}_n^{(t)}\right) &= -\int r \log q_{\widehat{\boldsymbol{\theta}}_n^{(t)}} \,\mathrm{d}\mu \\ &= -\int \frac{r}{q_{\widehat{\boldsymbol{\theta}}_n^{(t-1)}}} q_{\widehat{\boldsymbol{\theta}}_n^{(t-1)}} \log q_{\widehat{\boldsymbol{\theta}}_n^{(t)}} \,\mathrm{d}\mu \qquad \text{(S.12)} \\ &= -\mathbb{E}_{Q_{\widehat{\boldsymbol{\theta}}_n^{(t-1)}}}\left[h\left(\mathbf{X}, \widehat{\boldsymbol{\theta}}_n^{(t-1)}, \widehat{\boldsymbol{\theta}}_n^{(t)}\right)\right],\end{aligned}$$

where $\mathbf{X} \sim Q_{\widehat{\boldsymbol{\theta}}_n^{(t-1)}}$. The equation in (S.12) holds under the condition $Q^* \ll Q_{\boldsymbol{\theta}}$ for all $\boldsymbol{\theta} \in \boldsymbol{\Theta}_d$, because $q_{\widehat{\boldsymbol{\theta}}_n^{(t-1)}}(\mathbf{x}) = 0$ implies $r(\mathbf{x}) = 0$ for any $\mathbf{x}$.

We take a second-order expansion of $\mathcal{C}\left(\widehat{\boldsymbol{\theta}}_n^{(t)}\right)$ about $\boldsymbol{\theta}^*$ and use the

Taylor's remainder theorem:

$$\mathcal{C}\left(\widehat{\boldsymbol{\theta}}_n^{(t)}\right) = -\mathbb{E}_{Q_{\widehat{\boldsymbol{\theta}}_n^{(t-1)}}}\left[h\left(\mathbf{X}, \widehat{\boldsymbol{\theta}}_n^{(t-1)}, \boldsymbol{\theta}^*\right)\right] - \left(\widehat{\boldsymbol{\theta}}_n^{(t)} - \boldsymbol{\theta}^*\right)^\top \mathbb{E}_{Q_{\widehat{\boldsymbol{\theta}}_n^{(t-1)}}}\left[\nabla_{\boldsymbol{\theta}} h\left(\mathbf{X}, \widehat{\boldsymbol{\theta}}_n^{(t-1)}, \boldsymbol{\theta}^*\right)\right]$$
$$- \frac{1}{2}\left(\widehat{\boldsymbol{\theta}}_n^{(t)} - \boldsymbol{\theta}^*\right)^\top \mathbb{E}_{Q_{\widehat{\boldsymbol{\theta}}_n^{(t-1)}}}\left[\nabla^2_{\boldsymbol{\theta}} h\left(\mathbf{X}, \widehat{\boldsymbol{\theta}}_n^{(t-1)}, \boldsymbol{\theta}^*\right)\right]\left(\widehat{\boldsymbol{\theta}}_n^{(t)} - \boldsymbol{\theta}^*\right)$$
$$+ R_{4,n}, \tag{S.13}$$

where $R_{4,n}$ is the Taylor's remainder term at rate $\|\widehat{\boldsymbol{\theta}}_n^{(t)} - \boldsymbol{\theta}^*\|^3$ and has a bounded expectation of rate $\mathbb{E}(|R_{4,n}|) = O(\mathbb{E}\|\widehat{\boldsymbol{\theta}}_n^{(t)} - \boldsymbol{\theta}^*\|^3)$ due to assumption (B4) on the third derivatives. In (S.13), the zeroth-order term is $\mathcal{C}(\boldsymbol{\theta}^*)$ by definition and the first-order term is zero because

$$\mathbb{E}_{Q_{\widehat{\boldsymbol{\theta}}_n^{(t-1)}}}\left[\nabla_{\boldsymbol{\theta}} h\left(\mathbf{X}, \widehat{\boldsymbol{\theta}}_n^{(t-1)}, \boldsymbol{\theta}^*\right)\right] = \mathbb{E}_{Q_{\widehat{\boldsymbol{\theta}}_n^{(t-1)}}}\left[\frac{r(\mathbf{X})}{q_{\widehat{\boldsymbol{\theta}}_n^{(t-1)}}(\mathbf{X})}\nabla_{\boldsymbol{\theta}} \log q_{\boldsymbol{\theta}^*}(\mathbf{X})\right]$$
$$= \mathbb{E}_\mu\left[\frac{r(\mathbf{X})}{q_{\boldsymbol{\theta}^*}(\mathbf{X})}\nabla_{\boldsymbol{\theta}} q_{\boldsymbol{\theta}^*}(\mathbf{X})\right]$$
$$= \rho\mathbb{E}_\mu\left[\nabla_{\boldsymbol{\theta}} q_{\boldsymbol{\theta}^*}(\mathbf{X})\right] \tag{S.14}$$
$$= \rho\nabla_{\boldsymbol{\theta}}\mathbb{E}_\mu\left[q_{\boldsymbol{\theta}^*}(\mathbf{X})\right] \tag{S.15}$$
$$= 0,$$

where the equation in (S.14) holds under assumption (A1) and the interchange of expectation and differentiation in (S.15) holds under assumption (A6).

Let $\boldsymbol{\delta}_n := \sqrt{n}\left(\widehat{\boldsymbol{\theta}}_n^{(t)} - \boldsymbol{\theta}^*\right)$. Using the fact that $\mathbb{E}_{Q_{\widehat{\boldsymbol{\theta}}_n^{(t-1)}}}\left[h\left(\mathbf{X}, \widehat{\boldsymbol{\theta}}_n^{(t-1)}, \boldsymbol{\theta}^*\right)\right] = \mathcal{C}(\boldsymbol{\theta}^*)$ implies

$$\mathbb{E}_{Q_{\widehat{\boldsymbol{\theta}}_n^{(t-1)}}}\left[\nabla^2_{\boldsymbol{\theta}} h\left(\mathbf{X}, \widehat{\boldsymbol{\theta}}_n^{(t-1)}, \boldsymbol{\theta}^*\right)\right] = -\boldsymbol{\Gamma}$$

under assumption (A6), we simplify the expression of $\mathcal{C}\left(\widehat{\boldsymbol{\theta}}_n^{(t)}\right)$ in (S.13) as

$$\begin{aligned}\mathcal{C}\left(\widehat{\boldsymbol{\theta}}_n^{(t)}\right) &= \mathcal{C}(\boldsymbol{\theta}^*) - \frac{1}{2n}\boldsymbol{\delta}_n^\top \mathbb{E}_{Q_{\widehat{\boldsymbol{\theta}}_n^{(t-1)}}}\left[\nabla^2_{\boldsymbol{\theta}} h\left(\mathbf{X}, \widehat{\boldsymbol{\theta}}_n^{(t-1)}, \boldsymbol{\theta}^*\right)\right]\boldsymbol{\delta}_n + R_{4,n} \\ &= \mathcal{C}(\boldsymbol{\theta}^*) + \frac{1}{2n}\boldsymbol{\delta}_n^\top \boldsymbol{\Gamma}\boldsymbol{\delta}_n + R_{4,n}.\end{aligned}$$

Using Lemma 3, we can rewrite $\boldsymbol{\delta}_n$ as

$$\boldsymbol{\delta}_n = \sqrt{n}\left(\widehat{\boldsymbol{\theta}}_n^{(t)} - \boldsymbol{\theta}^*\right) = Z_{\widehat{\boldsymbol{\theta}}_n^{(t-1)}} + \epsilon_{n,\widehat{\boldsymbol{\theta}}_n^{(t-1)}},$$

where $E\|\epsilon_{n,\widehat{\boldsymbol{\theta}}_n^{(t-1)}}\| = o(1)$ since we have the uniform bound. And the covariance

$$\begin{aligned}\mathsf{Cov}(\boldsymbol{\delta}_n) &= \mathbb{E}\left(\mathsf{Cov}\left(\boldsymbol{\delta}_n|\widehat{\boldsymbol{\theta}}_n^{(t-1)}\right)\right) + \mathsf{Cov}\left(\mathbb{E}\left(\boldsymbol{\delta}_n|\widehat{\boldsymbol{\theta}}_n^{(t-1)}\right)\right) \\ &= \boldsymbol{\Gamma}^{-1}\mathbb{E}\left(\boldsymbol{\Lambda}_{\widehat{\boldsymbol{\theta}}_n^{(t-1)}}\right)\boldsymbol{\Gamma}^{-1} + o(1).\end{aligned} \tag{S.16}$$

Equation (S.16) is derived as follows. We first bound the quantity $\mathsf{Cov}\left(\mathbb{E}\left(\boldsymbol{\delta}_n|\widehat{\boldsymbol{\theta}}_n^{(t-1)}\right)\right)$. Note that for a random vector $\mathbf{X} = (X_1, X_2)$, its

covariance can be bounded by

$$
\begin{aligned}
|\mathsf{Cov}(X_1, X_2)| &\le \sqrt{\mathsf{Var}(X_1)\mathsf{Var}(X_2)} \\
&\le \max\{\mathsf{Var}(X_1), \mathsf{Var}(X_2)\} \\
&\le \max\{\mathbb{E}(X_1^2), \mathbb{E}(X_2^2)\} \\
&\le \mathbb{E}(\max\{X_1^2, X_2^2\}) \\
&\le \mathbb{E}(\|\mathbf{X}\|^2).
\end{aligned}
$$

The same bound can be established for a random vector of length $d$. By setting $\mathbf{X} = \mathbb{E}\Big(\epsilon_{n,\boldsymbol{\eta}=\widehat{\boldsymbol{\theta}}_n^{(t-1)}} | \widehat{\boldsymbol{\theta}}_n^{(t-1)}\Big)$ and using the fact that $\mathbb{E}\Big(Z_{\widehat{\boldsymbol{\theta}}_n^{(t-1)}} | \widehat{\boldsymbol{\theta}}_n^{(t-1)}\Big) = 0$, each element in the covariance matrix $\mathsf{Cov}\Big(\mathbb{E}\Big(\boldsymbol{\delta}_n | \widehat{\boldsymbol{\theta}}_n^{(t-1)}\Big)\Big) = \mathsf{Cov}\Big(\mathbb{E}\Big(\epsilon_{n,\boldsymbol{\eta}=\widehat{\boldsymbol{\theta}}_n^{(t-1)}} | \widehat{\boldsymbol{\theta}}_n^{(t-1)}\Big)\Big)$ is less than or equal to

$$
\begin{aligned}
\mathbb{E}\left\{\left\|\mathbb{E}\Big(\epsilon_{n,\widehat{\boldsymbol{\theta}}_n^{(t-1)}} \mid \widehat{\boldsymbol{\theta}}_n^{(t-1)}\Big)\right\|^2\right\} &\le \mathbb{E}\left\{\mathbb{E}^2\Big(\Big\|\epsilon_{n,\widehat{\boldsymbol{\theta}}_n^{(t-1)}}\Big\| \mid \widehat{\boldsymbol{\theta}}_n^{(t-1)}\Big)\right\} \\
&\le \sup_{\widehat{\boldsymbol{\theta}}_n^{(t-1)} \in \boldsymbol{\Theta}_d} \mathbb{E}^2\Big(\Big\|\epsilon_{n,\widehat{\boldsymbol{\theta}}_n^{(t-1)}}\Big\| \mid \widehat{\boldsymbol{\theta}}_n^{(t-1)}\Big) \\
&= \left(\sup_{\widehat{\boldsymbol{\theta}}_n^{(t-1)} \in \boldsymbol{\Theta}_d} \mathbb{E}\Big(\Big\|\epsilon_{n,\widehat{\boldsymbol{\theta}}_n^{(t-1)}}\Big\| \mid \widehat{\boldsymbol{\theta}}_n^{(t-1)}\Big)\right)^2 \\
&\le O(1/\sqrt{n}).
\end{aligned}
$$

Note that the last inequality is due to Lemma 3.

For another quantity, equation (S.11) implies

$$\mathbb{E}\left\|\boldsymbol{\Lambda}_{\widehat{\boldsymbol{\theta}}_n^{(t-1)}} - \rho\boldsymbol{\Gamma}\right\| = O\Big(\mathbb{E}\left\|\widehat{\boldsymbol{\theta}}_n^{(t-1)} - \boldsymbol{\theta}^*\right\|\Big) = o(1).$$

So we conclude

$$\mathsf{Cov}(\boldsymbol{\delta}_n) = \rho\boldsymbol{\Gamma}^{-1} + o(1). \tag{S.17}$$

This establishes the bound in equation (S.16).

Let

$$\tilde{\boldsymbol{\delta}}_n := \boldsymbol{\Gamma}^{1/2}\boldsymbol{\delta}_n = \sqrt{n}\boldsymbol{\Gamma}^{1/2}\left(\widehat{\boldsymbol{\theta}}_n^{(t)} - \boldsymbol{\theta}^*\right).$$

Then we have

$$\begin{aligned} \frac{1}{2n}\mathbb{E}\big[\boldsymbol{\delta}_n^\top\boldsymbol{\Gamma}\boldsymbol{\delta}_n\big] &= \frac{1}{2n}\mathbb{E}\Big[\tilde{\boldsymbol{\delta}}_n^\top\tilde{\boldsymbol{\delta}}_n\Big] \\ &= \frac{1}{2n}\mathsf{Tr}(\mathsf{Cov}(\tilde{\boldsymbol{\delta}}_n)) + o\Big(\frac{1}{n}\Big) \\ &= \frac{1}{2n}\mathsf{Tr}(\boldsymbol{\Gamma}^{1/2}\mathsf{Cov}(\boldsymbol{\delta}_n)\boldsymbol{\Gamma}^{1/2}) + o\Big(\frac{1}{n}\Big) \\ &= \frac{1}{2n}\rho d + o\Big(\frac{1}{n}\Big). \end{aligned} \tag{S.18}$$

As a result, we conclude that

$$\begin{aligned} \mathbb{E}\Big[\mathcal{C}\Big(\widehat{\boldsymbol{\theta}}_n^{(t)}\Big)\Big] &= \mathcal{C}(\boldsymbol{\theta}^*) + \frac{1}{2n}\mathbb{E}\big[\boldsymbol{\delta}_n^\top\boldsymbol{\Gamma}\boldsymbol{\delta}_n\big] + \mathbb{E}(R_{4,n}) \\ &= \mathcal{C}(\boldsymbol{\theta}^*) + \frac{\rho d}{2n} + o\Big(\frac{1}{n}\Big). \end{aligned} \tag{S.19}$$

**Analysis of the sample objective function $\bar{\mathcal{C}}_{\widehat{\boldsymbol{\theta}}_n^{(t-1)}}\left(\widehat{\boldsymbol{\theta}}_n^{(t)}\right)$.**

We take a second-order expansion of $\bar{\mathcal{C}}_{\widehat{\boldsymbol{\theta}}_n^{(t-1)}}\left(\widehat{\boldsymbol{\theta}}_n^{(t)}\right)$ about $\boldsymbol{\theta}^*$ with a Taylor remainder theorem on the third-order:

$$\begin{aligned}\bar{\mathcal{C}}_{\widehat{\boldsymbol{\theta}}_n^{(t-1)}}\left(\widehat{\boldsymbol{\theta}}_n^{(t)}\right) &= -\frac{1}{n}\sum_{i=1}^{n} h\left(\mathbf{X}_i^{(t-1)}, \widehat{\boldsymbol{\theta}}_n^{(t-1)}, \widehat{\boldsymbol{\theta}}_n^{(t)}\right) \\ &= -\frac{1}{n}\sum_{i=1}^{n}\left(h\left(\mathbf{X}_i^{(t-1)}, \widehat{\boldsymbol{\theta}}_n^{(t-1)}, \boldsymbol{\theta}^*\right) + \left(\widehat{\boldsymbol{\theta}}_n^{(t)} - \boldsymbol{\theta}^*\right)^\top \nabla_{\boldsymbol{\theta}} h\left(\mathbf{X}_i^{(t-1)}, \widehat{\boldsymbol{\theta}}_n^{(t-1)}, \boldsymbol{\theta}^*\right)\right. \\ &\quad \left. + \frac{1}{2}\left(\widehat{\boldsymbol{\theta}}_n^{(t)} - \boldsymbol{\theta}^*\right)^\top \nabla_{\boldsymbol{\theta}}^2 h\left(\mathbf{X}_i^{(t-1)}, \widehat{\boldsymbol{\theta}}_n^{(t-1)}, \boldsymbol{\theta}^*\right)\left(\widehat{\boldsymbol{\theta}}_n^{(t)} - \boldsymbol{\theta}^*\right)\right) \\ &\quad + R_{5,n} \qquad\qquad (\text{S}.20)\end{aligned}$$

where $R_{5,n}$ is the remainder of the expansion that involves $\|\widehat{\boldsymbol{\theta}}_n^{(t)} - \boldsymbol{\theta}^*\|^3$ and the third-order derivative of $h$. By assumption (B4), this quantity has an expectation $\mathbb{E}(|R_{5,n}|) = O(\mathbb{E}\|\widehat{\boldsymbol{\theta}}_n^{(t)} - \boldsymbol{\theta}^*\|^3)$. The zeroth-order term is $\bar{\mathcal{C}}_{\widehat{\boldsymbol{\theta}}_n^{(t-1)}}(\boldsymbol{\theta}^*)$ by definition. To re-express the first-order term, we use the fact

$$\nabla_{\boldsymbol{\theta}}\bar{\mathcal{C}}_{\widehat{\boldsymbol{\theta}}_n^{(t-1)}}\left(\widehat{\boldsymbol{\theta}}_n^{(t)}\right) = 0$$

or equivalently

$$\begin{aligned}0 &= \frac{1}{n}\sum_{i=1}^{n} \nabla_{\boldsymbol{\theta}} h\Big(\mathbf{X}_i^{(t-1)}, \widehat{\boldsymbol{\theta}}_n^{(t-1)}, \widehat{\boldsymbol{\theta}}_n^{(t)}\Big) \\ &= \frac{1}{n}\sum_{i=1}^{n} \nabla_{\boldsymbol{\theta}} h\Big(\mathbf{X}_i^{(t-1)}, \widehat{\boldsymbol{\theta}}_n^{(t-1)}, \boldsymbol{\theta}^*\Big) + \frac{1}{n}\sum_{i=1}^{n} \nabla^2_{\boldsymbol{\theta}} h\Big(\mathbf{X}_i^{(t-1)}, \widehat{\boldsymbol{\theta}}_n^{(t-1)}, \boldsymbol{\theta}^*\Big)\Big(\widehat{\boldsymbol{\theta}}_n^{(t)} - \boldsymbol{\theta}^*\Big) \\ &\quad + R_{6,n} \end{aligned} \tag{S.21}$$

where $R_{6,n}$ is another remainder of the expansion that involves $\|\widehat{\boldsymbol{\theta}}_n^{(t)} - \boldsymbol{\theta}^*\|^2$ and the third-order derivative of $h$. By assumption (B4), this quantity has an expectation $\mathbb{E}(|R_{6,n}|) = O(\mathbb{E}\|\widehat{\boldsymbol{\theta}}_n^{(t)} - \boldsymbol{\theta}^*\|^2)$.

Rearranging the equation in (S.21) yields

$$-\frac{1}{n}\sum_{i=1}^{n} \nabla_{\boldsymbol{\theta}} h\Big(\mathbf{X}_i^{(t-1)}, \widehat{\boldsymbol{\theta}}_n^{(t-1)}, \boldsymbol{\theta}^*\Big) = \frac{1}{n}\sum_{i=1}^{n} \nabla^2_{\boldsymbol{\theta}} h\Big(\mathbf{X}_i^{(t-1)}, \widehat{\boldsymbol{\theta}}_n^{(t-1)}, \boldsymbol{\theta}^*\Big)\Big(\widehat{\boldsymbol{\theta}}_n^{(t)} - \boldsymbol{\theta}^*\Big) + R_{6,n}.$$

Plugging this to the equation in (S.20) results in

$$\begin{aligned}\bar{\mathcal{C}}_{\widehat{\boldsymbol{\theta}}_n^{(t-1)}}\Big(\widehat{\boldsymbol{\theta}}_n^{(t)}\Big) &= -\frac{1}{n}\sum_{i=1}^{n}\Bigg( h\Big(\mathbf{X}_i^{(t-1)}, \widehat{\boldsymbol{\theta}}_n^{(t-1)}, \boldsymbol{\theta}^*\Big) + \Big(\widehat{\boldsymbol{\theta}}_n^{(t)} - \boldsymbol{\theta}^*\Big)^{\top} \nabla_{\boldsymbol{\theta}} h\Big(\mathbf{X}_i^{(t-1)}, \widehat{\boldsymbol{\theta}}_n^{(t-1)}, \boldsymbol{\theta}^*\Big) \\ &\qquad + \frac{1}{2}\Big(\widehat{\boldsymbol{\theta}}_n^{(t)} - \boldsymbol{\theta}^*\Big)^{\top} \nabla^2_{\boldsymbol{\theta}} h\Big(\mathbf{X}_i^{(t-1)}, \widehat{\boldsymbol{\theta}}_n^{(t-1)}, \boldsymbol{\theta}^*\Big)\Big(\widehat{\boldsymbol{\theta}}_n^{(t)} - \boldsymbol{\theta}^*\Big)\Bigg) + R_{5,n} \\ &= \bar{\mathcal{C}}_{\widehat{\boldsymbol{\theta}}_n^{(t-1)}}(\boldsymbol{\theta}^*) - \frac{1}{n}\boldsymbol{\delta}_n^{\top}\Bigg(-\frac{1}{n}\sum_{i=1}^{n} \nabla^2_{\boldsymbol{\theta}} h\Big(\mathbf{X}_i^{(t-1)}, \widehat{\boldsymbol{\theta}}_n^{(t-1)}, \boldsymbol{\theta}^*\Big)\Bigg)\boldsymbol{\delta}_n \\ &\qquad + \frac{1}{2n}\boldsymbol{\delta}_n^{\top}\Bigg(-\frac{1}{n}\sum_{i=1}^{n} \nabla^2_{\boldsymbol{\theta}} h\Big(\mathbf{X}_i^{(t-1)}, \widehat{\boldsymbol{\theta}}_n^{(t-1)}, \boldsymbol{\theta}^*\Big)\Bigg)\boldsymbol{\delta}_n + R_{5,n} + \Big(\widehat{\boldsymbol{\theta}}_n^{(t)} - \boldsymbol{\theta}^*\Big)^{\top} R_{6,n}.\end{aligned}$$

Let $\boldsymbol{\Gamma}_n\left(\widehat{\boldsymbol{\theta}}_n^{(t-1)}, \boldsymbol{\theta}^*\right) := -\frac{1}{n}\sum_{i=1}^n \nabla^2_{\boldsymbol{\theta}} h\left(\mathbf{X}_i^{(t-1)}, \widehat{\boldsymbol{\theta}}_n^{(t-1)}, \boldsymbol{\theta}^*\right)$. We can simplify the above equation as:

$$\begin{aligned}\bar{\mathcal{C}}_{\widehat{\boldsymbol{\theta}}_n^{(t-1)}}\left(\widehat{\boldsymbol{\theta}}_n^{(t)}\right) &= \bar{\mathcal{C}}_{\widehat{\boldsymbol{\theta}}_n^{(t-1)}}(\boldsymbol{\theta}^*) - \frac{1}{n}\boldsymbol{\delta}_n^\top \boldsymbol{\Gamma}_n\left(\widehat{\boldsymbol{\theta}}_n^{(t-1)}, \boldsymbol{\theta}^*\right)\boldsymbol{\delta}_n + \frac{1}{2n}\boldsymbol{\delta}_n^\top \boldsymbol{\Gamma}_n\left(\widehat{\boldsymbol{\theta}}_n^{(t-1)}, \boldsymbol{\theta}^*\right)\boldsymbol{\delta}_n \\ &\quad + R_{5,n} + \left(\widehat{\boldsymbol{\theta}}_n^{(t)} - \boldsymbol{\theta}^*\right)^\top R_{6,n} \\ &= \bar{\mathcal{C}}_{\widehat{\boldsymbol{\theta}}_n^{(t-1)}}(\boldsymbol{\theta}^*) - \frac{1}{2n}\boldsymbol{\delta}_n^\top \boldsymbol{\Gamma}_n\left(\widehat{\boldsymbol{\theta}}_n^{(t-1)}, \boldsymbol{\theta}^*\right)\boldsymbol{\delta}_n + R_{5,n} + \left(\widehat{\boldsymbol{\theta}}_n^{(t)} - \boldsymbol{\theta}^*\right)^\top R_{6,n}\end{aligned} \tag{S.22}$$

Note that the first term $\mathbb{E}\left[\bar{\mathcal{C}}_{\widehat{\boldsymbol{\theta}}_n^{(t-1)}}(\boldsymbol{\theta}^*)\right] = \mathcal{C}(\boldsymbol{\theta}^*)$ and by equation (S.4),

$$\boldsymbol{\Gamma}_n\left(\widehat{\boldsymbol{\theta}}_n^{(t-1)}, \boldsymbol{\theta}^*\right) = \boldsymbol{\Gamma} + \Delta_{n,\boldsymbol{\Gamma}}\left(\widehat{\boldsymbol{\theta}}_n^{(t-1)}\right),$$

where the later analysis in the proof of Lemma 3 showed that

$$\mathbb{E}\left(\left\|\Delta_{n,\boldsymbol{\Gamma}}\left(\widehat{\boldsymbol{\theta}}_n^{(t-1)}\right)\right\|\right) = O\left(\mathbb{E}\left(\left\|\widehat{\boldsymbol{\theta}}_n^{(t-1)} - \boldsymbol{\theta}^*\right\|\right)\right) = O(1/\sqrt{n}).$$

Thus, equation (S.18) yields that $\mathbb{E}\left(\boldsymbol{\delta}_n^\top \boldsymbol{\Gamma}_n\left(\widehat{\boldsymbol{\theta}}_n^{(t-1)}, \boldsymbol{\theta}^*\right)\boldsymbol{\delta}_n\right) = \rho d + o(1)$. So the expectation of equation (S.22) becomes

$$\mathbb{E}\left[\bar{\mathcal{C}}_{\widehat{\boldsymbol{\theta}}_n^{(t-1)}}\left(\widehat{\boldsymbol{\theta}}_n^{(t)}\right)\right] = \mathcal{C}(\boldsymbol{\theta}^*) - \frac{\rho d}{2n} + o\left(\frac{1}{n}\right). \tag{S.23}$$

Therefore, combining (S.19) and (S.23), the bias of interest is

$$\mathbb{E}\left[\bar{\mathcal{C}}_{\widehat{\boldsymbol{\theta}}_n^{(t-1)}}\left(\widehat{\boldsymbol{\theta}}_n^{(t)}\right) - \mathcal{C}\left(\widehat{\boldsymbol{\theta}}_n^{(t)}\right)\right] = \mathcal{C}(\boldsymbol{\theta}^*) - \frac{\rho d}{2n} - \left(\mathcal{C}(\boldsymbol{\theta}^*) + \frac{\rho d}{2n}\right) + o\left(\frac{1}{n}\right)$$
$$= -\frac{\rho d}{n} + o\left(\frac{1}{n}\right).$$

□

## Appendix B: EM Algorithm for Minimizing the Cross-Entropy

This appendix describes our version of the expectation-maximization (EM) algorithm to minimize the cross-entropy from a Gaussian mixture model (GMM) to a target distribution.

The GMM density is expressed as

$$q(\mathbf{x}; \boldsymbol{\theta}) = \sum_{j=1}^{k} \alpha_j \, q_j\left(\mathbf{x}; \boldsymbol{\mu}_j, \boldsymbol{\Sigma}_j\right), \tag{S.24}$$

where the component weights, $\alpha_j > 0, j = 1, \ldots, k$, satisfy $\sum_{j=1}^{k} \alpha_j = 1$. The $j$th Gaussian component density $q_j$ is parametrized by the mean $\boldsymbol{\mu}_j$ and the covariance $\boldsymbol{\Sigma}_j$. Thus, the model parameter $\boldsymbol{\theta}$ denotes $(\alpha_1, \ldots, \alpha_k, \boldsymbol{\mu}_1, \ldots, \boldsymbol{\mu}_k, \boldsymbol{\Sigma}_1, \ldots, \boldsymbol{\Sigma}_k)$.

To find the MCE $\widehat{\boldsymbol{\theta}}^{(t)}$ of $\boldsymbol{\theta}$, we want to minimize $\bar{\mathcal{C}}^{(t-1)}(\boldsymbol{\theta})$ in (16) and

thus set its gradient to zero:

$$-\frac{1}{\sum_{s=0}^{t-1} n_s} \sum_{s=0}^{t-1} \sum_{i=1}^{n_s} \nabla_{\boldsymbol{\theta}} h\Big(\mathbf{X}_i^{(s)}, \widehat{\boldsymbol{\theta}}^{(s)}, \boldsymbol{\theta}\Big) = 0.$$

This leads to the following updating equations for our version of the EM algorithm:

$$\alpha_j = \frac{\sum_{s=0}^{t-1} \sum_{i=1}^{n_s} \frac{r\left(\mathbf{X}_i^{(s)}\right)}{q_{\widehat{\boldsymbol{\theta}}^{(s)}}\left(\mathbf{X}_i^{(s)}\right)} \gamma_{ij}^{(s)}}{\sum_{s=0}^{t-1} \sum_{i=1}^{n_s} \frac{r\left(\mathbf{X}_i^{(s)}\right)}{q_{\widehat{\boldsymbol{\theta}}^{(s)}}\left(\mathbf{X}_i^{(s)}\right)}}, \tag{S.25}$$

$$\boldsymbol{\mu}_j = \frac{\sum_{s=0}^{t-1} \sum_{i=1}^{n_s} \frac{r\left(\mathbf{X}_i^{(s)}\right)}{q_{\widehat{\boldsymbol{\theta}}^{(s)}}\left(\mathbf{X}_i^{(s)}\right)} \gamma_{ij}^{(s)} \mathbf{X}_i^{(s)}}{\sum_{s=0}^{t-1} \sum_{i=1}^{n_s} \frac{r\left(\mathbf{X}_i^{(s)}\right)}{q_{\widehat{\boldsymbol{\theta}}^{(s)}}\left(\mathbf{X}_i^{(s)}\right)} \gamma_{ij}^{(s)}}, \tag{S.26}$$

$$\boldsymbol{\Sigma}_j = \frac{\sum_{s=0}^{t-1} \sum_{i=1}^{n_s} \frac{r\left(\mathbf{X}_i^{(s)}\right)}{q_{\widehat{\boldsymbol{\theta}}^{(s)}}\left(\mathbf{X}_i^{(s)}\right)} \gamma_{ij}^{(s)} (\mathbf{X}_i^{(s)} - \boldsymbol{\mu}_j)(\mathbf{X}_i^{(s)} - \boldsymbol{\mu}_j)^\top}{\sum_{s=0}^{t-1} \sum_{i=1}^{n_s} \frac{r\left(\mathbf{X}_i^{(s)}\right)}{q_{\widehat{\boldsymbol{\theta}}^{(s)}}\left(\mathbf{X}_i^{(s)}\right)} \gamma_{ij}^{(s)}}, \tag{S.27}$$

where

$$\gamma_{ij}^{(s)} = \frac{\alpha_j \, q_j\Big(\mathbf{X}_i^{(s)}; \boldsymbol{\mu}_j, \boldsymbol{\Sigma}_j\Big)}{\sum_{j'=1}^{k} \alpha_{j'} \, q_{j'}\Big(\mathbf{X}_i^{(s)}; \boldsymbol{\mu}_{j'}, \boldsymbol{\Sigma}_{j'}\Big)}. \tag{S.28}$$

The right-hand sides of the updating equations in (S.25), (S.26), and (S.27) involve

$$\boldsymbol{\theta} = (\alpha_1, \ldots, \alpha_k, \boldsymbol{\mu}_1, \ldots, \boldsymbol{\mu}_k, \boldsymbol{\Sigma}_1, \ldots, \boldsymbol{\Sigma}_k)$$

either explicitly or implicitly through $\gamma_{ij}^{(s)}$. Thus, the equations cannot

be analytically solved for $\boldsymbol{\theta}$. Instead, starting with an initial guess of $\boldsymbol{\theta}$, our version of the EM algorithm alternates between the expectation step (computing $\gamma_{ij}^{(s)}$) and the maximization step (updating $\boldsymbol{\theta}$) based on the updating equations until convergence is reached.

Prior studies [Botev et al., 2013, Wang and Zhou, 2015, Kurtz and Song, 2013] using mixture models for the cross-entropy method for importance sampling (recall that this method is a special case of the procedure in Figure 1 when $r$ is proportional to the optimal importance sampling density) do not iterate their updating equations; instead, they solve them only once when new data are gathered. This paper uses the aforementioned EM algorithm (i.e., iterating the updating equations until convergence) within the $t^{th}$ iteration to minimize $\bar{\mathcal{C}}^{(t-1)}(\boldsymbol{\theta})$ in (16). Note that the prior studies' one-step updating approach has a potential benefit of running a smaller risk of getting trapped in local minimizers. This is an active research topic due to its importance in stochastic gradient descent algorithms for training deep learning models.

### Updating Equations for Diagonal Covariance

In some situations it is advantageous to restrict the covariance matrix of each mixture component to be diagonal (or even isotropic). This can sub-

stantially reduce the number of free parameters for cases where the total sampling budget is small. For example, for fitting the diagonal covariance structure, we can derive an alternative updating equation for the covariance matrices by following the same derivation as with (S.27). This yields the following alternative updating equation:

$$\boldsymbol{\Sigma}_j = \frac{\sum_{s=0}^{t-1} \sum_{i=1}^{n_s} \frac{r\left(\mathbf{X}_i^{(s)}\right)}{q_{\widehat{\boldsymbol{\theta}}^{(s)}}\left(\mathbf{X}_i^{(s)}\right)} \gamma_{ij}^{(s)} \operatorname{diag}(\mathbf{X}_i^{(s)} - \boldsymbol{\mu}_j)^2}{\sum_{s=0}^{t-1} \sum_{i=1}^{n_s} \frac{r\left(\mathbf{X}_i^{(s)}\right)}{q_{\widehat{\boldsymbol{\theta}}^{(s)}}\left(\mathbf{X}_i^{(s)}\right)} \gamma_{ij}^{(s)}}. \tag{S.29}$$

## Appendix C: Numerical Experiments (Importance Sampling and Bayesian Inference)

This appendix presents the examples of using the CIC for approximating a) the optimal density for importance sampling and b) the posterior density in Bayesian inference. Each example evaluates the CIC's usefulness for each problem's purpose, i.e., a) minimizing the standard error of estimating a quantity of interest and b) sampling from an well-approximated posterior distribution, respectively, in comparison with similarly purposed methods. We refrain from using the population cross-entropy as a metric to evaluate the approximate distribution because 1) it has little practical meaning for

the BA problem where the computational cost of $r$ prohibits the metric calculation in practice and 2) even though it is calculated for toy examples, the unitless value of the cross-entropy makes its interpretation and meaningful comparison untenable.

**Case study: Structural safety analysis via importance sampling**

This subsection empirically demonstrates how the CIC can be used to approximate the optimal distribution of importance sampling for estimating $\rho$ in a classical structural safety problem [Kurtz and Song, 2013]. With $\mathbf{X}$ following the bivariate Gaussian density $\phi(\mathbf{x})$ with zero mean and identity covariance matrix, a system of interest fails when $\mathbf{X}$ falls on the region $\{\mathbf{x} \in \mathbb{R}^2 : g(\mathbf{x}) \leq 0\}$, where $g(\mathbf{x}) = b - x_2 - \kappa\,(x_1 - e)^2$. We vary the parameter $b = 1.5$, $2.0$, $2.5$ to test three different failure thresholds. We fix the other two parameters, $\kappa = 0.1$ and $e = 0$, to maintain the shape of the failure boundary $\{\mathbf{x} \in \mathbb{R}^2 : g(\mathbf{x}) = 0\}$ (the red dashed line in Figure 3). Note that $g(\mathbf{x})$ *represents* a computationally expensive function to evaluate, such as a finite element model in structural engineering.

The quantity of interest is the probability of the failure event, $\rho = \mathbb{E}_\mu[r]$, where $r(\mathbf{x}) = \phi(\mathbf{x})\mathbb{I}(g(\mathbf{x}) \leq 0)$. Here, $\mathbb{I}(\cdot)$ denotes the indicator function. We call the importance sampling method using the estimator $\widehat{\rho}^{(\tau)}$ in (19)

the *CIC-IS*. As benchmarks, we consider two other methods to estimate $\rho$. First, the crude Monte Carlo (CMC) estimator of $\rho$ is

$$\widehat{\rho}_{\mathrm{CMC}} = \frac{1}{n_{\mathrm{CMC}}} \sum_{i=1}^{n_{\mathrm{CMC}}} \mathbb{I}(g(\mathbf{X}_i) \le 0), \tag{S.30}$$

where $\mathbf{X}_i, i = 1, \ldots, n_{\mathrm{CMC}}$, are sampled from the density $\phi(\mathbf{x})$. Second, we implement the importance sampling method in Kurtz and Song [2013], which represents a state-of-the-art, cross-entropy method using a mixture model. Their method, which is called cross-entropy-based adaptive importance sampling using Gaussian mixture (CE-AIS-GM) uses the GMM with a *pre-specified* value for the number of mixture components, $k$. The GMM parameters are updated *once* within each iteration (as in Figure 5(b)) instead of using an EM algorithm.

For both importance sampling methods, we use the same sample size in Kurtz and Song [2013], namely, the total of 8700 replications: $n_t = 1000$ for $t = 0, \ldots, 6$ and $n_\tau = 1700$ for $\tau = 7$. Because the standard error of the CMC estimator can be analytically calculated as $\rho(1-\rho)/n_{\mathrm{CMC}}$, we estimate the standard error instead of running CMC simulations.

We set the CE-AIS-GM to use $k = 30$ and to estimate $\rho$ based only on the last ($\tau^{(th)}$) iteration data as in Kurtz and Song [2013]. The CIC-IS adaptively chooses $k$ within the algorithm described in Figure 4 and uses

the data from multiple iterations ($t = 2, \ldots, \tau$) to estimate $\rho$ by $\widehat{\rho}^{(\tau)}$ in (19), as described in Section 3.5. Because the CIC helps find a distribution fairly close to the optimal distribution throughout all the iterations, the CIC-IS can use the accumulated data to estimate $\rho$.

Table 1 shows the estimation results based on 500 experiment repetitions. As the parameter $b$ increases, the estimand $\rho$ decreases. Regardless of $b$, the CIC-IS obtains at least 50% smaller standard errors than the CE-AIS-GM. The smaller standard errors translate into larger computational savings for estimating $\rho$ at a desired accuracy. Specifically, to compare both importance sampling methods with CMC, we analytically calculate 'CMC Ratio', which is the number of replications used in each row's method (that is, 8700) divided by the number of replications necessary for the CMC estimator in (S.30) (that is, $n_{\text{CMC}}$) to achieve the same standard error in the row. Although CE-AIS-GM saves significantly compared to CMC, CIC-IS saves even more by 4 to 6 times. The good performance of the CIC-IS can be attributed to a) closeness of the approximate densities $q_{\widehat{\boldsymbol{\theta}}^{(t)}}$, $t = 1, \ldots, \tau$ to the optimal density $q^*$ as illustrated in Figure 3, and b) use of the data from multiple iterations ($t = 2, \ldots, \tau$) to estimate $\rho$, thanks to good approximate densities throughout the iterations.

Table 1: Comparison between CE-AIS-GM and CIC-IS

| $b$ | Method | Mean | Standard Error | CMC Ratio |
|---|---|---|---|---|
| 1.5 | CE-AIS-GM | 0.082902 | 0.001145 | 15.00% |
| | CIC-IS | 0.082911 | 0.000506 | 2.93% |
| 2.0 | CE-AIS-GM | 0.030174 | 0.000526 | 8.23% |
| | CIC-IS | 0.030173 | 0.000213 | 1.35% |
| 2.5 | CE-AIS-GM | 0.008908 | 0.000211 | 4.39% |
| | CIC-IS | 0.008910 | 0.000099 | 0.97% |

Note: 'Mean' and 'Standard Error' are the sample mean and standard error of the estimates, respectively. The 'CMC Ratio' is $n_{\text{Total}}/n_{\text{CMC}}$, where $n_{\text{Total}} = 8700$ and $n_{\text{CMC}} = \bar{\rho}(1-\bar{\rho})/(S.E.)^2$. $\bar{\rho}$ is the CIC-IS's sample mean and $S.E.$ is the standard error of the method in the row. The smaller the CMC Ratio, the larger the computational saving of the method over CMC.

## Example: Bayesian inference

The following two subsections visually demonstrate how the CIC can be used to approximate the posterior distribution in Bayesian inference.

### Simulation: Constrained linear regression

We consider an example where the coefficient vector $\boldsymbol{\beta}$ of a regression model is believed to lie in the unit square, i.e. $\boldsymbol{\beta} \in [0,1]^2$. To constrain our regression, we impose the following prior on $\boldsymbol{\beta}$, $f_0(\boldsymbol{\beta}) \propto \mathbf{1}\{\boldsymbol{\beta} \in [0,1]^2\} f(\boldsymbol{\beta})$, where $f(\boldsymbol{\beta})$ is the density of $\mathcal{N}(0, 3I_{2\times 2})$.

We generated a small synthetic dataset of 10 observations using the relation $Y_i = \boldsymbol{\beta}^\top X_i + \eta_i$, where $\boldsymbol{\beta} = [.6, 1]^\top$, $\mathbf{X}_i \overset{i.i.d}{\sim} \mathcal{N}(0, 3I_2)$, and $\eta_i \overset{i.i.d}{\sim} \mathcal{N}(0,1)$. We then used both the CIC (see Figure 4 and Appendix D for details) and the Metropolis-Hastings algorithm to approximate the posterior distribution, with each algorithm given a total sampling budget of $n = 10,000$.

For Metropolis-Hastings, the first 1000 samples were discarded to allow the Markov chain to reach stationarity. We used kernel density estimation with a Gaussian kernel to visualize the resulting density. The results in Figure S.1 show that both methods yielded similar posterior densities.

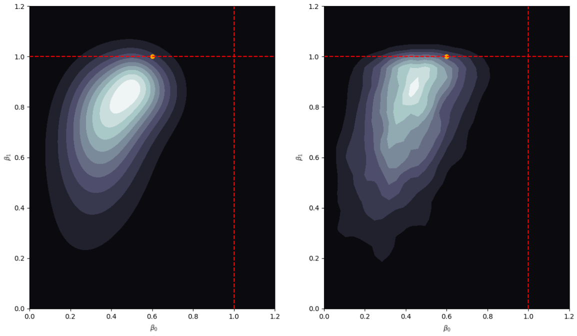

Figure S.1: Approximation of the posterior density using the CIC (left) and Metropolis-Hastings (right). Red dashed lines denote the boundary of the support for the true posterior density, i.e. the unit square. The orange dot indicates the location of $\boldsymbol{\beta} = [.6, 1]^\top$ which was used to generate the synthetic data of 10 observations.

**Case study: Inferring customers' shopping preferences**

We next consider a case study of inferring customers' shopping preferences from transaction data in Abreu [2011]. Each observation corresponds to a single transaction, where the predictors $\mathbf{X}_i \in \mathbb{R}^2$ are customer spend on the two grocery categories of fresh and frozen goods and the binary response $Y_i \in \{0, 1\}$ is the channel where the $i$-th transaction took place (retail location vs. convenience store). We wish to infer how customer propensity for one type of grocery is related to their choice of shopping venue.

We use a logistic model to capture the relationship between customer spend and their shopping location. In particular, we place the model $P(Y_i = 1) = \frac{1}{1+\exp(-s\boldsymbol{\beta}^\top \mathbf{X}_i)}$, where $\boldsymbol{\beta} \in [0,1]^2$ and $s > 0$ are two parameters. Note that we have an additional constraint that that $\beta_0 + \beta_1 = 1$ and $\beta_0, \beta_1 \geq 0$. $\boldsymbol{\beta}$ can be interpreted as the relative effect size between the fresh grocery versus the frozen grocery spending. The scale parameter $s$ reflects the overall effect of spending on the transaction place.

We impose a Dirichlet$([2,1]^\top)$ prior on $\boldsymbol{\beta}$ to reflect our belief that a higher propensity for customer spend on fresh groceries is associated with shopping in retail locations. We place a prior on $\log(s) \sim \mathcal{N}(\mu_s, .001)$. $\mu_s$ was chosen so that $s$ has a prior mean of 1.

Both the CIC and the Metropolis-Hastings algorithm were used to approximate the posterior distribution, with the same experimental conditions as the constrained linear regression example above.

The results in Figure S.2 show that the posterior approximations by the CIC and Metropolis-Hastings algorithms are dissimilar. We found that the underlying Markov chain was not mixing adequately in the $s$ parameter (see Figure S.3). This demonstrates how the CIC-based algorithm can approximate the posterior in a relatively automated manner, whereas manual diagnostics and tuning may be required to approximate the same posterior

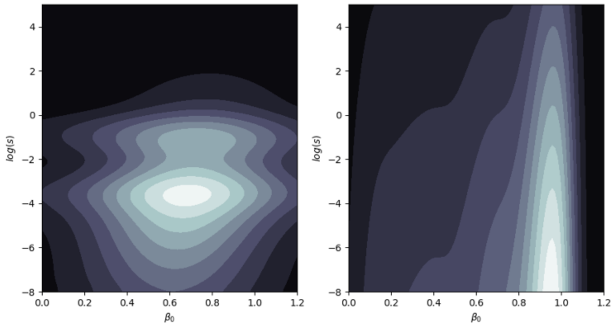


Figure S.2: Approximation of the posterior density using the CIC (left) and Metropolis-Hastings (right). Since the Dirichlet prior of $\boldsymbol{\beta} = (\beta_0, \beta_1)^\top$ implies that $\beta_0 + \beta_1 = 1$, we only plot the density of parameters $\beta_0$ and $s$.

distribution using MCMC methods such as Metropolis-Hastings.

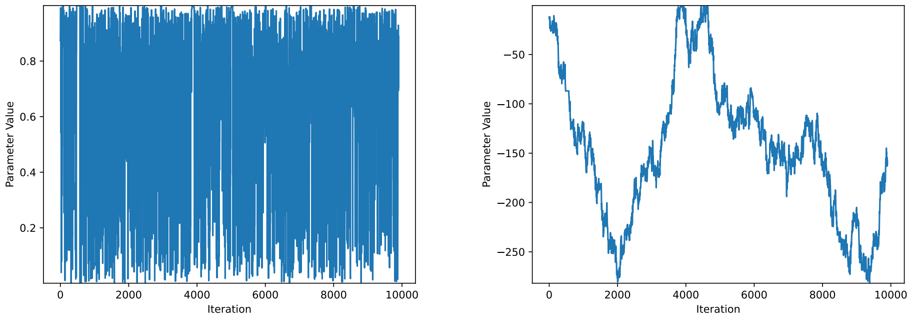


(a) $\beta_0$ parameter

(b) $s$ parameter

Figure S.3: Trace plots from the Metropolis-Hastings sampler for the $\beta_0$ and $s$ parameters. While the Markov chain appears to be well-mixing and stationary for the $\beta_0$ parameter, the trace plot for the $s$ parameter shows large autocorrelation and general non-stationarity.

# Appendix D: Implementation Details of Numerical Experiments

## Importance Sampling Example

This subsection describes the implementation details of the structural safety example in Appendix C to ensure the reproducibility. For the numerical experiment (with 500 repetitions), we randomly determined $\widehat{\boldsymbol{\theta}}^{(0)} = \boldsymbol{\eta}$ by drawing $\mu_1, \ldots, \mu_{30}$ from a standard multivariate Gaussian and setting all $\Sigma_1, \ldots, \Sigma_{30}$ as $3I_{p\times p}$.

For the implementation of the EM algorithm, we used multiple random initial values of $\boldsymbol{\theta}$ and chose the best minimizer of $\bar{\mathcal{C}}^{(t-1)}(\boldsymbol{\theta})$ in (16) to reduce the impact of initial guess of $\boldsymbol{\theta}$ on the algorithm's performance and avoid getting stuck with a local minimizer [Figueiredo and Jain, 2002]. In the $t^{th}$ iteration for $t \geq 1$, we randomly selected $\mu_1, \ldots, \mu_k$ from $\{\mathbf{X}_i^{(s-1)} : h\Big(\mathbf{X}_i^{(s-1)}, \widehat{\boldsymbol{\theta}}_n^{(s-1)}, \boldsymbol{\theta}\Big) > 0, i = 1, \ldots, n; s = 1, \ldots, t\}$ without replacement. However, if the set's cardinality was smaller than $k$, we randomly selected any elements in $\{\mathbf{X}_i^{(s-1)} : h\Big(\mathbf{X}_i^{(s-1)}, \widehat{\boldsymbol{\theta}}_n^{(s-1)}, \boldsymbol{\theta}\Big) = 0, i = 1, \ldots, n; s = 1, \ldots, t\}$ for the remaining parameters. We set $\Sigma_1, \ldots, \Sigma_k$ as $(3/p)\,\mathrm{trace}\big(cov(\bar{\mathbf{X}})\big)\, I_{p\times p}$, where $\bar{\mathbf{X}}$ is the data matrix created by augmenting $\{\mathbf{X}_i^{(s-1)} : i = 1, \ldots, n; s = 1, \ldots, t\}$, and $cov$ is the sample covariance. We used equal component

weights for the initialization, $\alpha_j = 1/k, j = 1, \ldots, k$. Note that when $k$ was updated, we reinitialized $\boldsymbol{\theta}$ as described above (i.e., cold start) because the updated $k$ changes the dimension of $\boldsymbol{\theta}$ and makes the use of previous iteration's $\boldsymbol{\theta}$ tricky in addition to the risk of getting stuck in the local minimizers found in the previous iteration. Future research may investigate how to leverage the previous iteration's information more intelligently (i.e., warm start) to enjoy better computational efficiency and more purposeful ways to avoid local minimizers.

When the number of components, $k$, became large enough to cause an overfitting issue within the EM algorithm, we caught it by monitoring the condition numbers of the Gaussian components' covariances [Figueiredo and Jain, 2002]. We aborted the EM algorithm when the condition number of any covariance exceeded $10^5$. If we needed to abort most of the EM algorithms that started with different initial parameter guesses (we used the threshold of 5 aborted out of 10), it indicated that $k$ is already too large for the given sample size.

To check the convergence of the EM algorithm, we checked the reduction of $\bar{\mathcal{C}}^{(t-1)}(\boldsymbol{\theta})$ in (16). We stopped iterating updating equations in the EM algorithm if the reduction of $\bar{\mathcal{C}}^{(t-1)}(\boldsymbol{\theta})$ was less than 1% or a specified maximum number of EM iterations, 10, is reached.

We computed the CIC for $k = k_{\min}, k_{\min}+1, \ldots, k_{\max}$ for the grid search of the minimizer $k^{*(t)}$ in the $t^{th}$ iteration. We set $k_{\min}$ as one for $t = 1$ and $\max\big(1, k^{*(t-1)} - 3\big)$ for $t \geq 2$. At $k = k_{\min}$, if all random initializations failed to converge, then we reduced $k_{\min}$ by one. In practice, it is generally unnecessary to increase $k$ up to $k_{\max}$, which is upper bounded by a known function of the sample size, because the overfitting is detected within the EM algorithm. To reduce the grid search time, we computed the moving average (with the window size of four) of the CIC and stopped increasing $k$ when the moving average started to increase.

**Bayesian Inference Examples**

For our implementation of Bayesian inference examples, we utilized *safe and effective importance sampling* (SEIS) [Owen and Zhou, 2000] to allow for this popular option to guard against the possibility of increased estimation variance. Note that the use of SEIS is entirely optional in our implementation although it seems generally beneficial in mitigating numerical issues in practice.

Specifically, we used deterministic mixture sampling in Owen and Zhou

[2000] with a mixture density

$$p_\alpha^{(t)} = \alpha q_{\widehat{\boldsymbol{\theta}}_n^{(t)}} + (1-\alpha)p,$$

where $p$ is the nominal density function and $\alpha \in [0,1]$. At each stage of our procedure, we perform deterministic mixture sampling: if a total of $n$ are to be drawn, we draw $[(1-\alpha)n]$ samples from $p$ and the remaining samples from $q_{\widehat{\boldsymbol{\theta}}_n^{(t)}}$. Any computations in our procedure where the density $q_{\widehat{\boldsymbol{\theta}}_n^{(t)}}$ would be used, we instead substitute $p_\alpha^{(t)}$. This includes calculation of $\bar{\mathcal{C}}_{\boldsymbol{\eta}}(\boldsymbol{\theta})$ as well as the EM procedure described in Appendix B. Additionally, we used the importance sampling estimator

$$\widehat{\rho}^{(t-1)} = \frac{1}{\sum_{s=1}^{t-1} n_s} \sum_{s=1}^{t-1} \sum_{i=1}^{n_s} \frac{r\left(\mathbf{X}_i^{(s)}\right) - \beta_1 q_{\widehat{\boldsymbol{\theta}}_n^{(t)}}\left(\mathbf{X}_i^{(s)}\right) - \beta_2 p\left(\mathbf{X}_i^{(s)}\right)}{p_\alpha^{(t)}\left(\mathbf{X}_i^{(s)}\right)} + \beta_1 + \beta_2$$

in place of (19). The control variate vector $\boldsymbol{\beta} = (\beta_1, \beta_2)^\top$ is computed using a least squares procedure which interested readers can learn about in Owen and Zhou [2000].

# Bibliography

Nuno Gonçalo Costa Fernandes Marques de Abreu. *Analise do perfil do cliente Recheio e desenvolvimento de um sistema promocional*. PhD thesis, 2011.

Rajendra J. Bhansali and Fredos Papangelou. Convergence of moments of least squares estimators for the coefficients of an autoregressive process of unknown order. *The Annals of Statistics*, 19(3):1155–1162, 1991.

Zdravko I. Botev, Dirk P. Kroese, Reuven Y. Rubinstein, and Pierre L'Ecuyer. The cross-entropy method for optimization. *Machine Learning: Theory and Applications, V. Govindaraju and C. R. Rao, Eds, Chennai: Elsevier*, 31:35–59, 2013.

Gerda Claeskens and Fabrizio Consentino. Variable selection with incomplete covariate data. *Biometrics*, 64(4):1062–1069, 2008. doi: 10.1111/j.1541-0420.2008.01003.x.

Michael C. Donohue, Rosanna Overholser, Ronghui Xu, and Florin Vaida. Conditional Akaike information under generalized linear and proportional hazards mixed models. *Biometrika*, 98(3):685–700, 2011.

Mario A. T. Figueiredo and Anil K. Jain. Unsupervised learning of finite

mixture models. *IEEE Transactions on Pattern Analysis and Machine Intelligence*, 24(3):381–396, 2002.

David F. Findley and Ching-Zong Wei. AIC, overfitting principles, and the boundedness of moments of inverse matrices for vector autotregressions and related models. *Journal of Multivariate Analysis*, 83(2):415–450, 2002.

Nolan Kurtz and Junho Song. Cross-entropy-based adaptive importance sampling using gaussian mixture. *Structural Safety*, 42:35–44, 2013.

Art Owen and Yi Zhou. Safe and effective importance sampling. *Journal of the American Statistical Association*, 95(449):135–143, 2000. ISSN 01621459. URL `http://www.jstor.org/stable/2669533`.

Reuven Y. Rubinstein and Alexander Shapiro. *Discrete event systems: Sensitivity analysis and stochastic optimization by the score function method.* Chichester: John Wiley & Sons Ltd, 1993.

Aad W. Van der Vaart. *Asymptotic statistics.* New York: Cambridge University Press, 1998.

Hui Wang and Xiang Zhou. A cross-entropy scheme for mixtures. *ACM Transactions on Modeling and Computer Simulation*, 25(1):6:1–6:20, 2015.
