# Supplementary material for: Information criterion for approximation of unnormalized densities
Source: PLoS One. 2025 Mar 17;20(3):e0317430. doi: 10.1371/journal.pone.0317430 (PMC11913297; doi:10.1371/journal.pone.0317430)
Supplement: S2 Additional experiments — It includes additional numerical experiments that investigate the empirical performance of the CIC-based importance sampling through two numerical examples and one case study (PDF) S3 Code. The code for reproducing all experimental results in the paper is publicly available as a Python package on https://pypi.org/project/cicriterion/. Its archived version’s DOI is doi: 10.5281/zenodo.13901261 [file pone.0317430.s002.pdf]

Information Criterion for Approximation of Unnormalized Densities

S2

Additional Experiments

To further investigate the empirical performance of the CIC-IS, we provide two numerical studies and a case study. The two numerical studies use specially constructed random events to test the ability of the CIC-IS to approximate optimal importance sampling (IS) distributions with both smooth, rounded supports and supports with sharp corners and linear boundaries (see Fig. 1). The case study uses the CIC-IS to estimate the probability of a cascading blackout in an electrical power grid, where the blackout represents a multi-dimensional random event with a combinatorial structure complicating the optimal IS distribution's support. The support's dimension (i.e., dimension of $\mathbf{X}$) is denoted by d throughout this supplement document and in our codebase (which abuses the notation used for the parameter dimension in the main text).

S1 Ball Probability Test

Our first experiment aims to approximate $\rho = P(||\mathbf{X}||^2 \leq R)$, where $\mathbf{X} \sim \mathcal{N}(0, I_{d\times d})$ and $R > 0$ is chosen so that ρ represents a small probability (close to 5%). Since $||\mathbf{X}||^2 \sim \chi^2_2(d)$, we can numerically compute ρ for comparison. For each value of d, we used the CIC-IS to estimate ρ with a total sampling budget of $n = 20,000$. This estimation was replicated 100 times. We report the sample mean and standard error of the 100 estimates. We consider the estimator to be biased if the true probability does not lie in the 95% confidence interval for ρ. The results can be seen in Table 1. As expected, the ball-shaped support of the optimal IS density allows the GMM-based approximation of CIC-IS to work well. The CIC-IS estimator appears to be unbiased and maintains the CMC ratio below 100% across d.

Table 1: Ball Test Results

d	R	ρ	Mean	Standard Error	MSE	CMC Ratio	Biased?
2	.100	0.0488	0.0488	0.000516	2.65×10^{-7}	11.46%	No
3	.346	0.0488	0.0488	0.000911	8.23×10^{-7}	35.77%	No
4	.700	0.0487	0.0487	0.000687	4.67×10^{-7}	20.36%	No

Note: 'Mean' and 'Standard Error' are the sample mean and standard error of the 100 estimates from the CIC-IS, respectively. The 'CMC Ratio' is $n_{\text{Total}}/n_{\text{CMC}}$, where $n_{\text{Total}} = 20000$ and $n_{\text{CMC}} = \bar{\rho}(1-\bar{\rho})/(S.E.)^2$. $\bar{\rho}$ is the sample mean and $S.E.$ is the standard error in the row. The smaller the CMC Ratio, the larger the computational saving of CIC-IS over CMC.

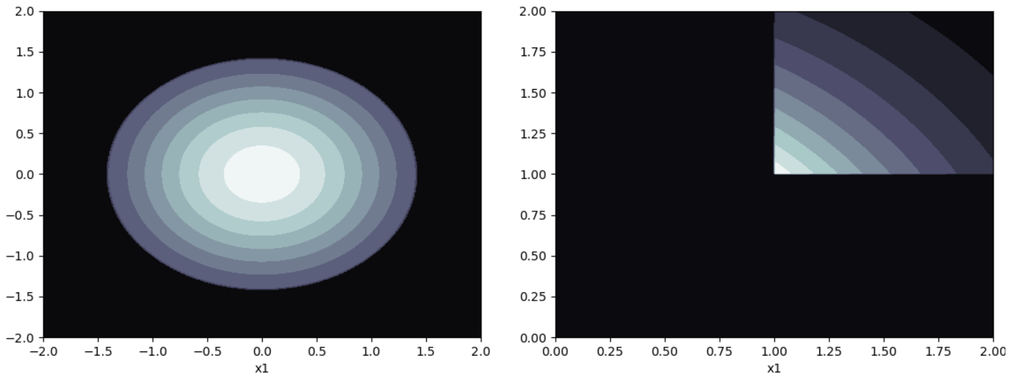

(a) Ball Test

(b) Tail Test

Figure 1: Examples of optimal IS densities for the ball test and tail test in two dimensions. Note that the optimal IS density for the ball test has a smooth boundary which can intuitively be easily approximated by a Gaussian mixture. On the other hand, the optimal IS density for the tail test has linear boundaries and a sharp corner at $\mathbf{x} = (1, 1)$.

S2 Tail Probability Test

Our second experiment uses the CIC-IS to estimate $\rho = P(\mathbf{X} \in [R, \infty)^d)$, where $\mathbf{X} \sim \mathcal{N}(0, I_{d\times d})$ and $R > 0$ is chosen so that ρ is small (around $[.003, .004]$). This test requires that the CIC-IS approximate the optimal IS density which has a support with a sharp corner and is more challenging to approximate than the ball test in Section S1. Table 2 shows the tail probability test result under the same experimental conditions as the ball test.

Note that the tests with $d = 3, 4$ resulted in CMC ratios greater than 100%, indicating that CIC-IS no longer saves computing resources over CMC. For $d = 4$, the estimator is even biased, indicating a fundamental failure of the IS algorithm. We attribute these characteristics to 1) the sharp boundaries of the optimal IS density's support and 2) the fact that the number of edges which form the sharp boundary increases with d, further complicating the geometry of the optimal IS density's support, thereby making the GMM-based approximation increasingly challenging.

Table 2: Tail Probability Test Results

d	R	ρ	Mean	Standard Error	MSE	CMC Ratio	Biased?
2	1.6	0.00300	0.00300	5.87×10^{-5}	3.41×10^{-9}	1.15%	No
3	1	0.00399	0.00405	0.00138	1.88×10^{-6}	941.28%	No
4	.7	0.00343	0.000603	0.00251	1.42×10^{-5}	20818.44%	Yes

Note: See the note of Table 1 for the interpretation of this table.

S3 Power Grid Failure Case Study

This case study uses the CIC-IS to estimate the probability of a cascading blackout in a real electric power grid containing 42 electrical relays. The power grid model is a default model provided in the COSMIC cascading power outage simulator [Song et al., 2015]. Due to the fact that power grids are generally designed to be robust to a single component failure,

a property known as $N-1$ reliability [Poyrazoglu and Oh, 2015], it is necessary to model at least two component failures for a cascading blackout to be possible.

For our experiments, we select a subset of d electrical relays and model the relative time-to-failure of each relay as an independent standard normal random variable. Any components with a relative time-to-failure less than $-R$ are forced to fail in the COSMIC power grid simulation and the binary outcome of the simulation is recorded. More precisely, the outcome of the event can be written as $h(\mathbf{X}) = H(\{i : X_i < -R\})$, where $\mathbf{X} = (X_1, \ldots, X_d)$, and $H(E)$ is a map from the set of failed electrical relay indices $E \subset 2^{\{1,2,\ldots,42\}}$ to the binary outcome of the deterministic COSMIC simulator.

Note that the structure of this case study is similar to the tail probability test in Section S2, with the caveat that the event of interest may not occur in some regions of the tail support. This makes the case study a similarly hard problem as that in the tail probability test. The regions of the tail where the event occurs are determined by the combinatorial structure of the power grid. By enumerating all possible combinations, we analytically derived the probability ρ of a cascading blackout in Table 3 to evaluate the CIC-IS. Note that in practice, ρ, is typically an unknown estimand that

cannot be analytically derived.

Table 3 shows the case study results under the same experimental conditions as the previous two tests in Sections S1 and S2. The results indicate that CIC-IS does not save computing resources over CMC (i.e., CMC Ratio is greater than 100%) for all d's. The CMC Ratio worsens more slowly than the exponentially increasing d, presumably because of the peculiar structure of the simulation's event of interest (e.g., a cascading blackout occurs once a few critical electrical relays out of d selected ones fail). Even for the largest d, CIC-IS yielded an unbiased estimator, showing some robustness in covering the support of the optimal IS density in the case study. Regardless, the optimal IS density seems to take a too complex shape to approximate well using the GMM-based CIC-IS in this case study.

Table 3: Case Study Results

d	R	ρ	Mean	Standard Error	MSE	CMC Ratio	Biased?
3	1	0.02523	0.02520	0.001256	1.56×10^{-6}	128.55%	No
5	1.2	0.03272	0.03256	0.001732	3.00×10^{-6}	190.50%	No
10	1.3	0.03382	0.03391	0.005436	2.93×10^{-5}	1108.64%	No
20	1.3	0.03322	0.03356	0.004917	2.40×10^{-5}	1490.91%	No
42	1.3	0.03371	0.03357	0.005383	2.87×10^{-5}	1786.16%	No

Note: See the note of Table 1 for the interpretation of this table.

Bibliography

Gokturk Poyrazoglu and HyungSeon Oh. Optimal topology control with physical power flow constraints and N-1 contingency criterion. *IEEE Transactions on Power Systems*, 30(6):3063–3071, 2015. doi: 10.1109/TPWRS.2014.2379112.

Jiajia Song, Eduardo Cotilla-Sanchez, Goodarz Ghanavati, and Paul DH Hines. Dynamic modeling of cascading failure in power systems. *IEEE Transactions on Power Systems*, 31(3):2085–2095, 2015.
